# Efficient Removal of Tetracyclines and Their Metabolites from Wastewater Using Purified Stevensite: Adsorption Capacity, Reusability, and Antibiotic Decontamination

**DOI:** 10.3390/antibiotics14040395

**Published:** 2025-04-10

**Authors:** Noelia García-Criado, Laura Martín-Pozo, Julia Martín, Juan Luis Santos, Irene Aparicio, Esteban Alonso

**Affiliations:** Departamento de Química Analítica, Escuela Politécnica Superior, Universidad de Sevilla, E-41011 Seville, Spain; ngarcia5@us.es (N.G.-C.); lpozo@us.es (L.M.-P.); jlsantos@us.es (J.L.S.); iaparicio@us.es (I.A.); ealonso@us.es (E.A.)

**Keywords:** tetracyclines, antibiotics, removal, clay, absorption, environmental water

## Abstract

**Background/Objectives:** The persistence of tetracycline residues in aquatic environments poses substantial risks to ecosystems and public health, emphasizing the need for effective removal strategies. This study examines the use of purified stevensite (ST), a natural clay mineral, as an efficient and cost-effective adsorbent for removing tetracycline antibiotics from contaminated water. **Methods:** Batch experiments were conducted to assess the adsorption kinetics, isotherms, and influence of environmental factors. Material characterization studies were performed before and after tetracycline adsorption. **Results:** ST demonstrated optimal removal efficiency at an acidic pH, achieving over 99% elimination of both tetracyclines and their metabolites at an adsorbent dose of 2 g L^−1^ and antibiotic concentration of 5 mg L^−1^. Equilibrium was reached within 30 min. Regeneration experiments confirmed that ST retained over 90% of its adsorption capacity after five adsorption–desorption cycles. Surface characterization revealed that ST’s large surface area, high cation exchange capacity, and potential for hydrogen bonding may explain its high adsorption capabilities. The material was tested on real samples of tap water, surface water, and wastewater, demonstrating an effective removal rate over 99%. **Conclusions:** With its high efficiency, low cost and favourable reusability, purified ST is a promising option for large-scale wastewater treatment, contributing to safer water resources and improved environmental protection.

## 1. Introduction

Antibiotics have been extensively used in clinical therapy, aquaculture, and animal husbandry due to their broad-spectrum antibacterial properties and low production costs. One of the main concerns regarding the release of antibiotics into aquatic environments is the generation of antimicrobial resistance genes and antimicrobial-resistant bacteria [1,2]. Among these, tetracyclines rank among the most extensively used worldwide. Alongside many antibiotics, tetracyclines have been classified as emerging contaminants by the United States Environmental Protection Agency (USEPA) and the European Union (EU) due to their increasing prevalence in surface water, groundwater, drinking water, and wastewater effluents [2,3]. Both tetracyclines and their derivatives are highly resistant to biodegradation processes and are difficult for humans and animals to metabolize. After ingestion, most tetracyclines are excreted intact through urine and faeces [4], entering water and soil systems in unmetabolized forms, which significantly contribute to environmental pollution. As a result, tetracycline concentrations in aquatic systems have risen dramatically over recent decades. Many studies have reported tetracycline concentrations ranging from ng L^−1^ to µg L^−1^ in various aquatic systems, including lakes, rivers, and surface and groundwaters, with particularly high levels observed in raw and treated sewage [2,3]. Concentrations as high as mg L^−1^ have also been detected in influent outflows near pharmaceutical wastewater treatment plants [5]. Tetracycline antibiotics in aquatic environments can undergo a series of reactions, resulting in degradation products and the formation of tetracycline derivatives that may exhibit higher or lower toxicity than their parent compounds [6]. In addition, residual antibiotics and their derivatives can further promote the prevalence of antibiotic-resistant genes and bacteria, posing serious threats to aquatic ecosystems and public health [1,2].

Various techniques have been proposed for the removal of tetracycline antibiotics from aqueous solutions, including adsorption, membrane filtration, coagulation and flocculation, electrochemical methods, biological treatments, and advanced oxidation processes [7,8,9,10]. Among these, adsorption is widely regarded as one of the most effective and cost-efficient techniques for tetracycline removal from wastewater due to its simplicity, reusability, and high efficiency [10,11]. Several adsorbent materials, such as carbon nanotubes and ion-exchange resins, have been investigated for antibiotic removal from wastewater [12]. However, their high adsorption coefficients contrast with their high production and operating costs [12]. Consequently, cost-effective alternatives such as clays have gained attention for their antibiotic adsorption potential [13,14,15]. Natural clays are increasingly used for contaminant adsorption due to their high surface area, low cost, and abundance in soils. Clay minerals such as montmorillonite, kaolinite, bentonite, and rectorite, among others, have been utilized for this purpose, with or without surface modifications [14,15]. This study proposes the removal of tetracycline antibiotics and their metabolites through adsorption onto stevensite (ST), a clay mineral member of the montmorillonite group. ST is a trioctahedral Mg-rich smectite with a layer charge derived from vacant octahedral positions. The mineral exhibits a large specific surface area, exceptional ion exchange capacities, and swelling properties, making it invaluable for a wide range of applications [13,15]. Unlike other smectites such as montmorillonite, ST is a trioctahedral clay with a high swelling capacity, exceptional surface area, and substantial cation exchange capability, all of which enhance its effectiveness for antibiotic adsorption. Its octahedral vacancies reduce the structural charge, favouring interactions with polar molecules such as tetracyclines and their metabolites. These properties have been extensively studied in the context of smectite applications for emerging contaminant removal in aqueous media [16]. However, natural ST may contain impurities such as quartz, feldspars, and iron oxides, which do not contribute to adsorption and may hinder its efficiency. To optimize its adsorptive performance, a purification step was carried out to remove these non-adsorptive mineral phases and isolate the finest fraction of ST. This process enhances the material’s specific surface area and cation exchange capacity, thereby facilitating interactions with tetracycline antibiotics and their metabolites. The removal of coarse particles and impurities further improves the adsorption efficiency, aligning with previous findings on the purification of clay minerals for environmental applications [16,17]. Antón-Herrero et al. (2018) studied the adsorption capacity of tetracycline (TC), oxytetracycline (OTC), and chlortetracycline (CTC) on commercial ST, demonstrating a high removal capacity from solution through displacement by cation exchange [16]. However, other possible adsorption mechanisms have also been observed, such as surface complexation or hydrogen bonding [18].

The aim of this study was to evaluate the adsorption capacity of commercial ST for the removal of commonly used tetracycline antibiotics along with their main metabolites from water. The analytes studied were eight tetracycline drugs (TC, CTC, minocycline (MNC), demeclocycline (DMCC), doxycycline (DC), OTC, tigecycline (TGC), and metacycline (MTC)) and five metabolites (4-epitetracycline (ETC), anhydrotetracycline (ATC), 4-epianhydrotetracycline (EATC), 4-epichlortetracycline (ECTC), and 4-epioxytetracycline (EOTC). To the best of our knowledge, this is the first study evaluating this clay for the removal of such a wide range of tetracycline antibiotics as well as their derived metabolites. Isotherm and kinetic studies were conducted, and the effects of pH, salinity, and the organic matter content on adsorption capacity were evaluated. The reusability of ST was assessed through five consecutive adsorption–desorption cycles. Additionally, the adsorption mechanism was explored by comparing the material’s characteristics before and after antibiotic adsorption. Finally, the applicability of ST was tested using real water samples, including tap water, surface water, effluent, and influent wastewater.

## 2. Results and Discussion

### 2.1. Adsorbent Characterization

The ST adsorbent was characterized before and after the batch experiments. ST is a naturally occurring clay recognized for its high crystallinity and large specific surface area. The N_2_ adsorption–desorption isotherm analysis revealed a BET surface area of 147 m^2^ g^−1^ and a pore volume of 0.122 cm^3^ g^−1^ (Table S1), which confirms its high adsorption potential. Following the desorption process, the specific surface area, pore size, and pore volume were reduced, while the high adsorption capacity was largely preserved. These results align with those reported in previous studies. For instance, Gharous et al. (2024) similarly reported a high surface area (147 m^2^ g^−1^) and a slightly lower pore volume of 0.088 cm^3^ g^−1^ [11]. Carvalho et al. (2022) synthesized ST using a simple method involving nitric and hydrochloric acids, obtaining two clay samples with BET surface areas of 163 m^2^ g^−1^ and 197 m^2^ g^−1^ and pore volumes of 0.93 cm^3^ g^−1^ and 1.09 cm^3^ g^−1^, respectively. These values are consistent with our findings, further supporting the high adsorption potential of ST-based materials [19].

A type II isotherm was obtained for ST (Figure S1). Type II isotherms exhibit a gradual convex upward curve shape at low concentrations, followed by a sharp concave upward trend [20]. The initial convex region corresponds to monolayer adsorption on the surface, indicating a material with accessible adsorption sites. At low relative pressures, nitrogen uptake is associated with micropore filling. As the pressure increases, multilayer adsorption occurs, transitioning from surface interaction to bulk adsorption. The sharp concave increase at higher pressures, is attributed to capillary condensation within mesopores, leading to a steep rise in the adsorption capacity. This type of isotherm is characteristic of mesoporous materials, indicating a well-defined pore structure, and is a trend commonly observed in layered silicates [20]. The average pore diameter of ST was found to be 3.32 nm, placing it within the mesoporous range (from 2 to 50 nm) according to the IUPAC definition [21].

**Figure 1:**
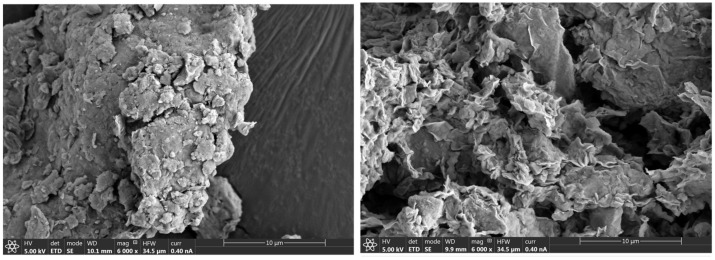
SEM characterization of ST before (**left panel**) and after (**right panel**) the adsorption of tetracycline antibiotics and their metabolites.

**Figure 2:**
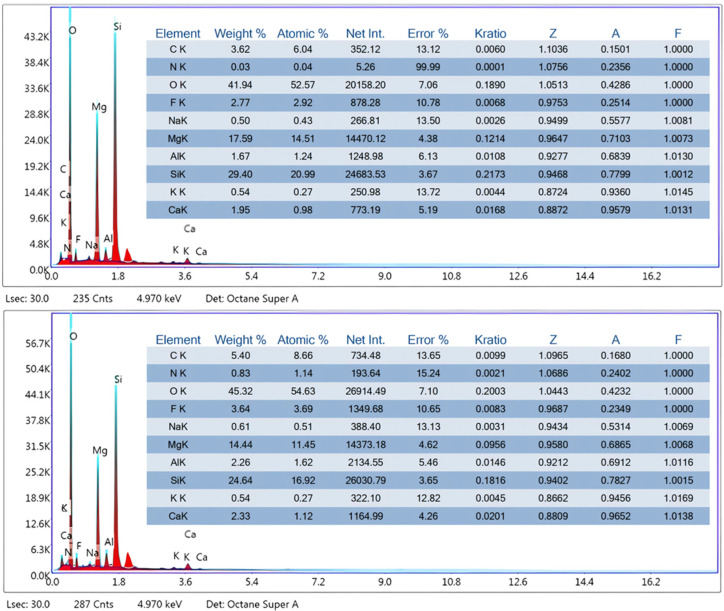
EDS characterization of ST before (**top panel**) and after (**bottom panel**) the adsorption of tetracycline antibiotics and their metabolites.

The surface morphology of ST was examined through scanning electron microscopy coupled with energy-dispersive X-ray spectroscopy (SEM/EDS) imaging (Figure 1, Figure 2 and Figure S2). The analysis showed the presence of agglomerated, irregularly shaped layers, consistent with smectite clay structures [22,23,24]. This morphology, characterized by the porous and layered nature of ST, suggests a high specific surface area and well-developed pore structure, providing abundant adsorption sites for pollutants and contributing to its efficiency as an adsorbent. After adsorption, the surface experienced a subtle increase in roughness, as observed in the images, indicating the interaction of tetracycline molecules with the material. This observation aligns with the results from the nitrogen adsorption–desorption isotherms. Both SEM and isotherm data support the mesoporous structure of ST and its potential effectiveness as an adsorbent for pollutant removal.

According to the EDS analysis, O, Si, Mg, and C were the predominant elements in the composition of ST, accounting for 41.94%, 29.4%, 17.49%, and 3.62% by weight, respectively. After the adsorption of tetracyclines, notable changes were observed in the elemental composition of the material. In particular, increases in the C, N, and O contents were detected, rising to 5.40%, 0.83%, and 45.32%, respectively. Since these elements are key structural components of tetracyclines, their increased presence on the surface of ST provides direct evidence of successful adsorption. In addition to these increases, the EDS analysis also revealed significant variations in the concentrations of native elements of the clay. Magnesium and silicon showed marked reductions, decreasing from 17.59% to 14.44% and from 29.40% to 24.64%, respectively. As these elements are major constituents of the ST structure, their decrease suggests partial coverage of the mineral surface by the adsorbed tetracycline molecules, which may reduce the detectability of these elements in the EDS spectrum due to the formation of an organic coating. Other elements such as aluminium and calcium experienced slight increases from 1.67% to 2.26% and from 1.95% to 2.33%, respectively, possibly indicating minor interactions or ion exchange processes between the clay and the tetracyclines. Fluorine also increased from 2.77% to 3.62%, which may reflect localized changes in surface chemistry following adsorption. These findings collectively support the occurrence of adsorption and surface modification of ST after contact with tetracyclines, as reflected by the incorporation of organic elements and the reduced contribution of key mineral components in the EDS analysis.

**Figure 3:**
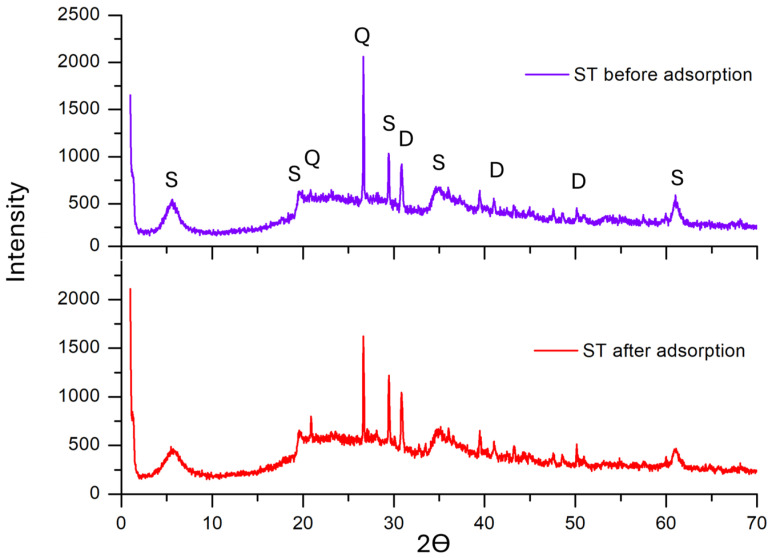
XRD patterns of ST before (**top panel**) and after (**bottom panel**) tetracycline adsorption. S—stevensite; D—dolomite; Q—quartz.

An X-ray diffraction (XRD) analysis (Figure 3) was performed to examine the crystalline structure and mineralogical composition of ST. The diffraction pattern displays distinct peaks at 1.00°, 5.69°, 19.59°, 29.49°, 35.08°, and 61.07°, which are characteristic of the smectite phase and have been previously reported for ST [11]. Additionally, peaks at 20.97° and 26.75° confirm the presence of quartz, while peaks at 30.97°, 40.98°, and 50.18° correspond to dolomite [25,26,27]. These results confirm the mineralogical composition of the ST sample and align with previously reported XRD data for ST and its associated mineral phases.

**Figure 4:**
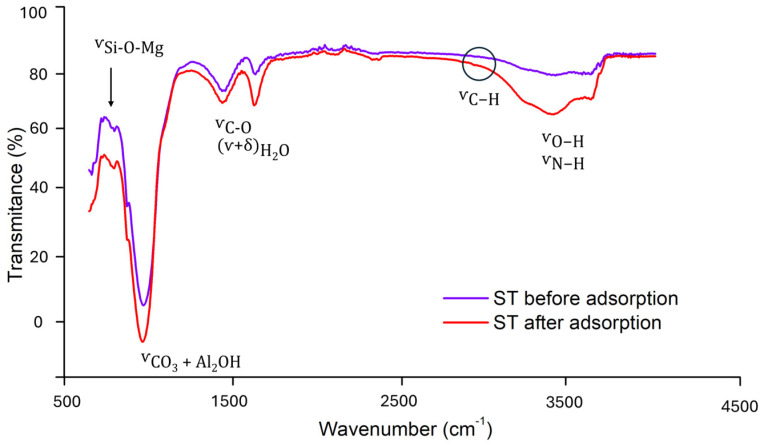
FTIR spectra of ST before and after tetracycline antibiotic adsorption.

The Fourier transform infrared (FTIR) spectra (Figure 4) provide deeper insights into the functional groups and chemical interactions within ST before and after tetracycline adsorption. In the raw clay sample, five prominent bands were identified, confirming the characteristic composition of ST. The high-frequency band at 3384.44 cm^−1^ corresponds to the stretching vibration of OH groups (ʋO-H), indicative of structural hydroxyls. The bands at 1639.70 cm^−1^ and 1442.39 cm^−1^ are attributed to H_2_O bending vibrations ((ʋ + δ)H_2_O) and carbonate (CO_3_^2−^) stretching, respectively. At lower frequencies, the peak at 973.26 cm^−1^ is assigned to CO_3_^2−^ and Al_2_OH groups, while the band at 795.27 cm^−1^ corresponds to the Si-O-Mg bond, characteristic of ST [28].

Upon tetracycline adsorption, the FTIR spectrum reveals that the primary ST bands remain largely unchanged, aligning with previous studies on smectite clays [29] and indicating that the overall clay structure is not significantly altered. However, significant spectral changes are observed, particularly in the regions around 970, 1440, and 1640 cm^−1^, where an increase in intensity suggests contributions from adsorbed tetracycline antibiotics. The band at ~1640 cm^−1^, initially assigned to H_2_O bending vibrations, intensifies due to overlap with the stretching vibration of the C=O bond in tetracycline rings, while the band at ~1440 cm^−1^, attributed to carbonate (CO_3_^2−^), also increases in intensity due to the additional contribution of the C=C skeletal vibrations. Similarly, the peak at ~970 cm^−1^, originally linked to CO_3_^2−^ and Al_2_OH groups, exhibits an increase in intensity, suggesting further interactions between these antibiotics and the mineral surface.

Additionally, a notable broadening and intensification of the bands around 3400 and 3600 cm^−1^ is observed, indicating enhanced hydrogen bonding interactions. This suggests that the adsorption of tetracyclines is not solely governed by cation exchange but also involves interactions with structural hydroxyl groups and water molecules within the clay. These findings reinforce the idea that tetracycline adsorption occurs through multiple mechanisms, including hydrogen bonding and coordination with interlayer cations, as previously reported in studies on smectites and biochar–clay composites [27,29].

This behaviour, combined with its high surface area, mesoporous structure, and stable mineral composition, reinforce its potential as an efficient and reusable adsorbent for pollutant removal.

### 2.2. Adsorption Studies

#### 2.2.1. Kinetic Studies

**Figure 5:**
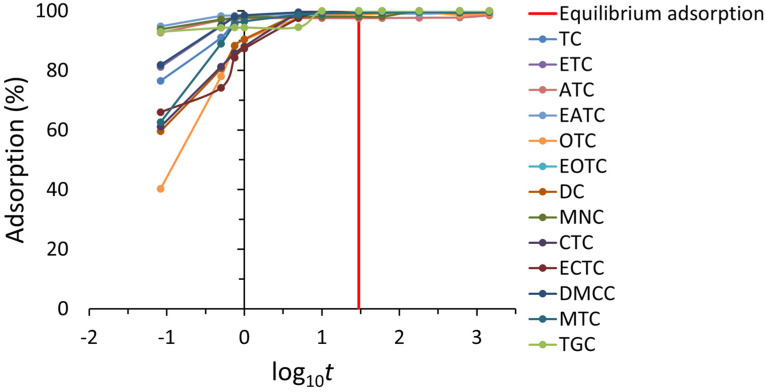
Adsorption kinetics of tetracyclines onto ST (adsorption vs. log10 time), with the equilibrium time (30 min) highlighted by a red line.

The kinetic studies were conducted under the following conditions: contact time of 0.083–1440 min, adsorbent dosage of 0.02 g per 10 mL, and antibiotic solution concentration of 2 mg mL^−1^. These conditions applied in the batch experiments, are detailed in Figure S4. The results are presented in Figure 5, where the adsorption equilibrium can be clearly observed. The adsorption rate of tetracycline antibiotics onto ST was remarkably high. Within the first 0.5 min, an adsorption efficiency exceeding 80% was observed for most compounds, and equilibrium was reached for all selected tetracyclines in less than 5 min. Once equilibrium was established, no further changes in the adsorption capacity were detected over time. However, a contact time of 30 min was selected for subsequent experiments to ensure complete adsorption.

The mechanism controlling the adsorption process was examined using pseudo-first-order (PFO) and pseudo-second-order (PSO) kinetic models, as shown in Table S2. The PFO model assumes that the adsorption rate is proportional to the available adsorption sites on the surface and physical diffusion is the rate-limiting step (physical adsorption). The PSO model relates the adsorption rate to the squares of the unoccupied adsorption sites on the surface, involving chemical interactions (chemical adsorption) [11]. The rate constants for the adsorption of tetracyclines onto ST were determined graphically (Figure S5) and the experimental data obtained are summarized in Table 1. The PSO model provided the best fit for the experimental data, as indicated by its higher correlation coefficient, which was close to unity for all tetracycline compounds (>0.99) (Figure S6). Furthermore, the experimental q_e_ values closely matched the calculated q_e_ values, confirming the reliability of the model. These findings align with previous studies that have used clay-based adsorbents for antibiotic removal. Several studies have reported that the pseudo-second-order (PSO) model provides the best fit for tetracycline adsorption onto clay-based materials. For instance, Turan et al. (2022) investigated the removal of TC using zirconium-loaded chitosan modified with perlite, reporting that the adsorption kinetics followed the PSO model, with equilibrium reached in approximately 6–7 h [30]. Similarly, Maged et al. (2019) evaluated the adsorption of TC, CTC, and OTC onto bentonite and found that the PSO model best described the kinetic data [31]. Li et al. (2021) studied the adsorption of TC onto Eu/Zr-MOF and observed that the PSO model provided the best fit, with equilibrium attained within 2.5 h [32]. Zhao et al. (2011) reported that approximately 80% of tetracycline sorption onto goethite occurred within the first 4 h. Based on this observation, an equilibration time of 24 h was selected for their experiments. The adsorption kinetics of tetracycline onto goethite were best described by the pseudo-second-order model [33]. Gharous (2024) also demonstrated that the adsorption of TC and CTC onto methionine-modified ST followed the PSO kinetic model, with rapid adsorption occurring within the first hour [11]. The results of the present study align with these findings, yet they also highlight a distinctive aspect: the adsorption process onto ST was significantly faster, achieving equilibrium within just 5 min. This remarkably short equilibrium time suggests that ST could be a highly efficient adsorbent for tetracyclines removal.

#### 2.2.2. Isotherm Studies

**Figure 6:**
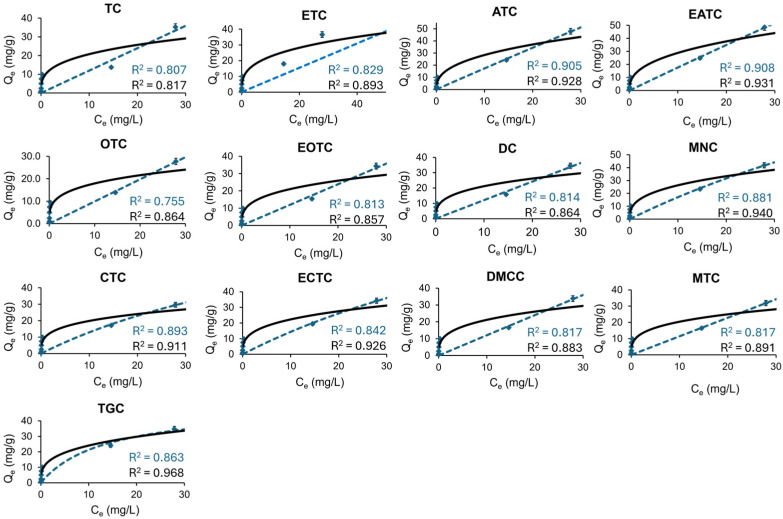
Isotherm data (points), Langmuir model (dashed blue line), and Freundlich model (solid black line) for the adsorption of tetracycline and its metabolites onto ST. R^2^ values from the non-linear fitting of each model are shown.

In this study, the adsorption of tetracyclines onto ST was evaluated across concentrations ranging from 1 to 100 mg L^−1^, significantly exceeding (2000 times higher) the typical environmental levels [3,5,6]. The quantity of tetracycline adsorbed (q_e_) as a function of its equilibrium concentration (C_e_) is presented in Figure 6.

The adsorption data were analysed using Langmuir and Freundlich isotherm models (Table S2). The Langmuir isotherm assumes monolayer adsorption onto a homogeneous surface with a finite number of identical adsorption sites, whereas the Freundlich isotherm model accounts for multilayer adsorption on heterogeneous surfaces, where the adsorption varies from one site to another. Figure 6 shows the graphs obtained. The parameters derived from these models are summarized in Table 2. In all cases, the Freundlich model provided a better fit (0.817 < R^2^ < 0.968) compared to the Langmuir model (0.755 < R^2^ < 0.908). This suggests that the adsorption process of tetracyclines onto the ST surface occurs on heterogeneous sites with varying adsorption energies. Another parameter to consider is the Freundlich parameter n. A Freundlich parameter *n* > 1 indicates favourable adsorption conditions, whereas an *n* < 1 suggests less favourable conditions at higher concentrations [34]. The Freundlich parameter *n* was found to be less than one (0.288 < *n* < 0.389), indicating that while adsorption is more efficient at lower concentrations, the efficiency decreases as concentration increases [34]. Despite this, high removal efficiencies were observed, over 97% at 20 mg L^−1^ and above 60% at 100 mg L^−1^, demonstrating the adsorbent’s effectiveness even at higher concentrations. These findings align with those of Parolo et al. (2008), who studied the adsorption of tetracycline on montmorillonite and found that the Freundlich model adequately described the process, indicating adsorption on heterogeneous surfaces. In this study, despite *n* values that were below one, the adsorption process remained highly effective, particularly at environmentally relevant concentrations [35].

On the other hand, the Langmuir model also showed a reasonable fit to the data (0.755 < R^2^ < 0.908). The maximum monolayer adsorption capacity (q_max_) obtained from the Langmuir model was high, ranging from 86.06 mg g^−1^ for TGC to over 200,000 mg g^−1^ for tetracycline, following the order TGC > ECTC > MNC > CTC > MTC > DMCC > ATC > ETC > EATC > OTC > EOTC > DC > TC. These high q_max_ values suggest that although adsorption occurs on a heterogeneous surface (as indicated by the Freundlich fit), the adsorbent also exhibits high monolayer adsorption capacity for tetracyclines, possibly due to strong interactions at specific active sites. This dual behaviour is consistent with the findings of previous studies. For instance, Filho et al. (2023) reported an adsorption capacity of 178.65 mg g^−1^ for tetracycline onto a chitosan–alginate–bentonite composite, with adsorption best described by the Langmuir model, suggesting that monolayer adsorption can dominate under certain conditions. Similarly, other studies have shown that tetracyclines can exhibit both multilayer adsorption at lower concentrations (Freundlich model) and monolayer saturation at higher concentrations (Langmuir model), depending on the surface properties of the adsorbent [36].

These results reinforce the idea that tetracycline adsorption onto clay is a complex process influenced by surface heterogeneity, the adsorption site distribution, and concentration effects. The consistency of our findings with previous research highlights the relevance of both Freundlich and Langmuir models in describing the adsorption behaviour onto clay materials, depending on the prevailing conditions.

#### 2.2.3. Influence of Environmental Conditions: pH, Salt, and Organic Content

**Figure 7:**
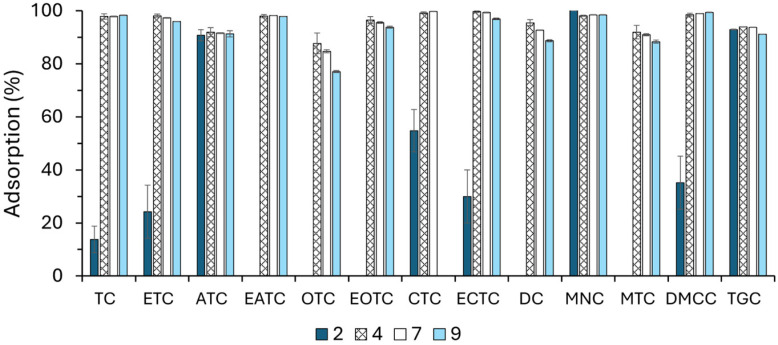
Effect of pH on the adsorption (%) of tetracyclines on ST.

pH plays a crucial role in the adsorption of tetracyclines onto solid surfaces, as it directly affects both the ionization state of the antibiotics and the surface charge of the adsorbent. Tetracyclines exhibit three dissociation constants (pKa ≈ 3.3, 7.7, and 9.6), meaning they exist in different ionic forms depending on the pH of the solution (H_3_TC^+^ at pH < 3.3, H_2_TC^0^ between pH 3.3 and 7.7, and HTC− or TC^2−^ at pH > 7.7) [35]. Similarly, the surface charge of the ST adsorbent varies with the pH, being positively charged at low pH due to protonation and negatively charged at high pH due to deprotonation, which influences electrostatic interactions with the antibiotic molecules. As shown in Figure 7, the adsorption efficiency of ST remained over 99% for most tetracyclines over a wide pH range, except under extreme conditions. A significant reduction in adsorption was observed at very acidic pH (pH 2) for most compounds, except for ATC, MNC, and TGC. This decline can be attributed to two main factors: (i) the repulsion between the protonated tetracycline molecules (H_2_TC^+^) and the positively charged ST surface, and (ii) the degradation of certain tetracyclines under highly acidic conditions, as reported in previous studies [13]. As can be seen in Figure 7, MTC, DC, ATC, and EATC may have degraded at pH 2, while at higher pH (9), the degradation of TGC, OTC, ECTC, and CTC may have occurred, albeit to a lesser extent. This behaviour is likely explained by the low stability of certain tetracyclines in acidic environments and the electrostatic repulsion between their anionic forms and the negatively charged ST surface at high pH. A general decrease in the adsorption capacity was observed as pH increased to 9, consistent with previous findings on the adsorption of tetracyclines onto clay-based materials [37]. The highest adsorption occurred in the pH 3–6 range, where tetracyclines predominantly exist in their zwitterionic form (H_2_TC^0^), which favours electrostatic attraction with the moderately charged ST surface. At pH > 7.7, the dominance of anionic species (HTC− and TC^2−^) results in repulsion from the negatively charged adsorbent, leading to a decline in adsorption efficiency [35,36]. Beyond electrostatic interactions, additional mechanisms, such as complexation with surface functional groups, hydrogen bonding, and hydrophobic interactions, may also play a role in the adsorption process, depending on the specific properties of the adsorbent and environmental conditions [33].

Dissolved organic matter (DOM) has been shown to play a crucial role in adsorption processes. Particles of organic matter, such as humic or fulvic acids, can compete with the active sites of the adsorbent material for the binding of pollutants, affecting the adsorption rate [11]. Therefore, the influence of DOM on the adsorption of tetracyclines onto ST was evaluated using humic acid solutions (0 to 25 mg L^−1^) at a constant tetracycline mixture concentration (2 mg mL^−1^). As demonstrated in Figure S7, the adsorption capacity remained consistent across all concentrations, indicating a minimal effect of these variables on the removal efficiency. The results suggest that the structural and surface characteristics of ST provide stable adsorption sites that are not significantly disrupted by the presence of humic substances. This stability may be attributed to the dominant role of electrostatic interactions and specific surface binding mechanisms that are less affected by organic matter competition. Although DOM is known to influence adsorption processes by interacting with contaminants or adsorbent surfaces, in this case, its presence did not lead to noticeable variations in adsorption efficiency. However, slight differences were observed for OTC and its metabolite EOTC. OTC exhibited a small, progressive reduction in adsorption efficiency between 0 and 3% as DOM concentration increased from 0 to 25 mg L^−1^, while EOTC showed an even smaller decrease of approximately 0–1.5%. These minor reductions suggest that, for these specific compounds, DOM could introduce a weak competitive effect or subtle modifications in molecular interactions at the adsorption sites. In contrast, the adsorption efficiency of all other tetracyclines remained nearly unaffected, consistently maintaining values over 99%, regardless of the DOM concentration. These findings align with previous studies on clay-based adsorbents, which have reported that the adsorption of tetracyclines remains largely stable in the presence of humic substances due to strong surface interactions [3,33].

The progressive reduction in the adsorption efficiency observed with increasing NaCl concentrations suggests that ionic strength plays a significant role in the adsorption behaviour of tetracyclines and their metabolites onto ST (Figure S8). As the NaCl concentration increased from 0% to 4% *v*/*v*, all compounds exhibited a decline in adsorption capacity. Among the tested compounds, OTC was the most affected, with a 50% reduction in removal efficiency, maintaining an adsorption efficiency of approximately 50% across all salt concentrations. This was followed by its metabolite, EOTC, which showed a slightly less pronounced decline, with adsorption decreasing by 30–40%. This suggests that while both OTC and its metabolite are susceptible to increased salinity, the parent compound is more significantly impacted. Similarly, TC, ETC, ATC, and ECTC experienced moderate declines of around 20%, reducing their adsorption efficiency from 99% to approximately 80%. Notably, some of their metabolites, such as ECTC, exhibited slightly greater stability under saline conditions compared to their parent compounds. Meanwhile, EATC, CTC, ECTC, MNC, and DMCC exhibited a more moderate decline of approximately 10%, demonstrating a clear trend of decreasing adsorption efficiency with increasing salinity.

This suggests that for these compounds, increasing the ionic strength progressively reduces their interactions with the adsorbent, likely due to enhanced competition with Na^+^ ions or changes in their molecular conformation that affect the binding affinity. The reduction in the adsorption efficiency can be attributed to two primary mechanisms: competition for adsorption sites and complexation between Na^+^ ions and tetracycline molecules. Sodium ions may compete directly with tetracycline species for binding sites on the ST surface, effectively reducing the number of available active sites for antibiotic adsorption. Additionally, sodium ions are known to form soluble complexes with tetracyclines, altering their molecular structure and charge distribution [37]. This complexation may reduce the electrostatic attraction between tetracyclines and the negatively charged adsorption sites of ST, thereby limiting the adsorption process [36]. Furthermore, an increased ionic strength can lead to compression of the electrical double layer, reducing the accessibility of tetracycline molecules to adsorption sites [35]. Similar trends have been reported in previous studies investigating the effect of the ionic strength on tetracycline adsorption. For example, Maged et al. (2019) observed a decrease in TMP adsorption efficiency due to the competition between Na^+^ ions and antibiotic molecules, a phenomenon consistent with the current findings [31]. These results suggest that the adsorption efficiency of tetracyclines onto ST is highly dependent on the ionic composition of the solution, which should be carefully considered in environmental applications where variable salinity conditions may affect the removal performance.

### 2.3. Mechanistic Study

**Figure 8:**
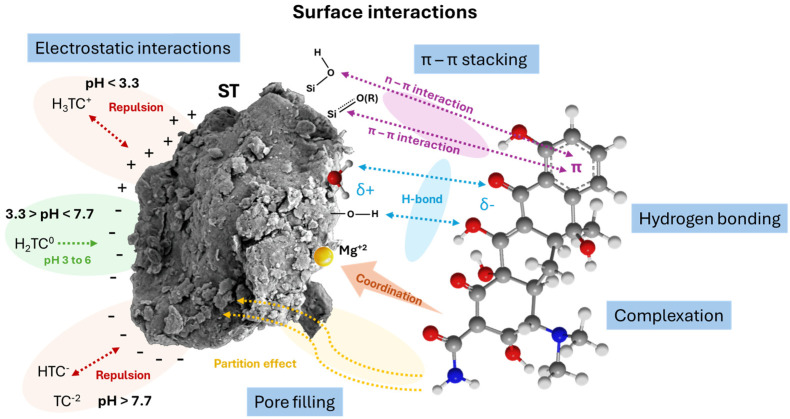
Proposed mechanism for tetracycline antibiotic adsorption on ST.

The adsorption process is inherently complex and influenced by multiple factors, such as the nature of the adsorbate, the physicochemical properties of the adsorbent, and the characteristics of the aqueous medium. Figure 8 shows the proposed adsorption mechanism. According to the EDS analysis (Figure 2), an increase in the elemental composition of C, N, and O was observed after adsorption, rising from 3.62% to 5.40%, 0.03% to 0.83%, and 41.94% to 45.32%, respectively. Since these elements are key structural components of tetracyclines, their increased presence on the surface of ST provides direct evidence of successful adsorption. Further structural confirmation was attempted through an XRD analysis; however, no significant changes were observed in the diffraction patterns after adsorption (Figure 3). This indicates that the adsorption process does not alter the crystalline structure of ST, suggesting that tetracycline retention occurs primarily through surface interactions rather than intercalation or structural modifications, as previously reported for smectite clays [35].

To further investigate the adsorption mechanism, FTIR spectroscopy was used to compare the vibrational bands of the adsorbent before and after the interaction with tetracycline antibiotics (Figure 4). The adsorption process resulted in notable shifts in both the peak intensity and position, indicating the presence of molecular interactions between the adsorbent and the antibiotics. Specifically, two prominent peaks were detected post-adsorption: one at 1600 cm^−1^, corresponding to H_2_O stretching and bending vibrations ((ʋ + δ)H_2_O), and another at 1370 cm^−1^, attributed to the carbonyl (CO–) functional group. These spectral changes confirm that tetracyclines interact with the ST surface through various adsorption mechanisms. The rapid initial adsorption phase suggests that electrostatic interactions play a crucial role in binding positively charged tetracyclines to the negatively charged ST surface. However, given the stability of the XRD pattern, these interactions occur predominantly at the surface rather than within the interlayer spaces of the clay [16].

This hypothesis is further supported by zeta potential measurements (Table S1), which indicate changes in the surface charge of the adsorbent upon antibiotic adsorption. Initially, ST exhibited a negative zeta potential of −8.7 mV at pH 3.43, confirming its negatively charged surface under these conditions. After antibiotic adsorption, the zeta potential remained negative at −8.14 mV, suggesting that electrostatic interactions were not the only driving force in the adsorption process. The minimal change in zeta potential indicates that tetracycline adsorption does not significantly alter the overall surface charge of ST, reinforcing the idea that other interactions, such as hydrogen bonding and π-π interactions, play a dominant role [13].

Beyond electrostatic forces, hydrogen bonding is likely another key contributor to the adsorption process. The carboxyl and amine functional groups present in the ST structure provide active sites for dipole interactions with tetracyclines. In the 3700–2500 cm^−1^ region, characteristic O–H and N–H vibrational bands were detected, both of which are influenced by hydrogen bonding [29]. After adsorption, the intensity of these bands decreased (Figure 4), suggesting the formation of intermolecular hydrogen bonds between the O–H and N–H groups of ST and tetracyclines. Additionally, π-π interactions may also contribute to adsorption, as the Si-O-Si bonds in the ST structure could facilitate stacking interactions with the aromatic rings of tetracyclines [37].

Another possible mechanism is pore filling, where the porous structure of ST allows tetracycline antibiotics to diffuse into and occupy the available voids. The high surface area and mesoporosity of ST make it an effective adsorbent for the removal of tetracycline antibiotics and their metabolites. The material exhibited a pore size of 3.32 nm and a pore volume of 0.12 cm^3^ g^−1^, providing sufficient space for the adsorption process. Notably, after adsorption, the specific surface area and pore volume underwent significant reductions, decreasing from 147 to 111 m^2^ g^−1^ and from 0.12 to 0.09 cm^3^ g^−1^ (Table S1). This decrease could be due to the saturation of the adsorption sites as they become occupied by tetracycline molecules, which would lead to a reduction in the pore size and, consequently, a decrease in the specific surface area. These morphological changes were also evident in the SEM images post-adsorption (Figure S3). The observed decline indicates that tetracycline molecules successfully occupied the available adsorption sites, further confirming pore filling as a contributing mechanism in the adsorption process [21].

Considering all the analytical evidence, a multifaceted physisorption mechanism is proposed for ST–tetracycline interactions. This includes electrostatic attraction, hydrogen bonding, π-π interactions, and pore filling, all of which play roles in the adsorption process. The stability of the XRD pattern after adsorption suggests that these mechanisms occur at the surface rather than inducing structural changes within ST [13,35]. The combined effect of these mechanisms highlights the strong affinity between ST and tetracyclines, demonstrating its potential for effective antibiotic removal in aqueous systems.

### 2.4. Desorption and Reuse

The reusability of an adsorbent is a critical factor in determining its economic viability and practical application in water treatment. In this study, the desorption efficiency of tetracyclines from ST was evaluated over five consecutive cycles (Figure S9). After each adsorption cycle, the ST sample was treated with 5 mL of a mixture composed of McIlvaine buffer and acetone (7:3, *v*/*v*) at pH 4, and then washed with deionized water. The desorption mechanism involves both ion exchange and solvent-mediated desorption processes. It has been observed that acidic eluents such as 0.1% formic acid (pH 2.6) generate a high concentration of hydronium ions (H_3_O^+^), which weaken the electrostatic interactions between the adsorbed tetracyclines and the functional groups of the adsorbent [12]. This process facilitates the release of antibiotics through ion exchange mechanisms, enhancing the desorption efficiency. Meanwhile, acetone, a small polar solvent, can penetrate the interlayer spaces of the adsorbent, interact with functional groups of the tetracyclines, and promote desorption. Similar solvent-assisted desorption strategies have been effectively applied in the removal of antibiotics from solid matrices, demonstrating their efficiency in regenerating adsorbents for multiple reuse cycles [31,32]. The cycling experiments revealed that all tetracyclines could be desorbed with an efficiency exceeding 99% after four consecutive cycles, confirming the high regeneration potential of the adsorbent. After the fifth reuse cycle, the adsorption efficiency decreased slightly for three tetracyclines (OTC, EOTC, and TGC), remaining above 90% (decrease of 10%). The ability to maintain a high adsorption capacity after repeated use can be attributed to its high surface area and porous structure, as demonstrated by the characterization studies (Table S1), which enhance the availability of active sites. The structural stability of ST also minimizes degradation after regeneration. Moreover, morphological and surface analyses confirmed that the adsorbent experienced minimal alterations after a desorption cycle (Figure S3), ensuring its continued effectiveness in tetracycline removal. Antón-Herrero et al. (2018) studied the desorption of three tetracyclines (OTC, TC, and CTC) from ST and found that the desorption ratio was lower than the adsorption ratio, indicating the occurrence of hysteresis. They concluded that the irreversible adsorption of a fraction of the tetracycline molecules could take place within the clay structure [16]. This phenomenon could explain the slight decrease in adsorption efficiency observed after the fifth cycle. The outstanding regeneration capability is a crucial advantage, as it significantly enhances the cost-effectiveness and sustainability of the material in large-scale applications. Efficient regeneration not only extends the lifespan of the adsorbent but also minimizes secondary waste generation, which is essential for environmentally friendly wastewater treatment technologies.

### 2.5. Adsorption in Real Environmental Matrices

The presence of tetracyclines in the environment has been widely reported in various studies [3]. For instance, Lenart-Boroń et al. (2022) reported tetracycline concentrations of up to 1.7 µg L^−1^ in surface water [38]. Antos et al. (2024) reviewed the occurrence and fate of tetracyclines in European aquatic environments and found that average contamination levels ranged from 0 to 20 ng/L over the past decade [3]. Moreover, studies of agricultural soils and manure indicate that tetracyclines can persist for extended periods, with concentrations depending on the livestock type and environmental conditions [3]. Therefore, the use of simulated systems becomes essential to evaluate the real-world applicability of the adsorption process. To enhance environmental relevance, adsorption assays were conducted using fortified environmental water samples, including influent and effluent wastewater, surface water, and tap water. Prior to analysis, the samples were filtered through a 1.2 μm glass microfiber membrane filter. Quantification was performed using matrix-matched calibration curves.

**Figure 9:**
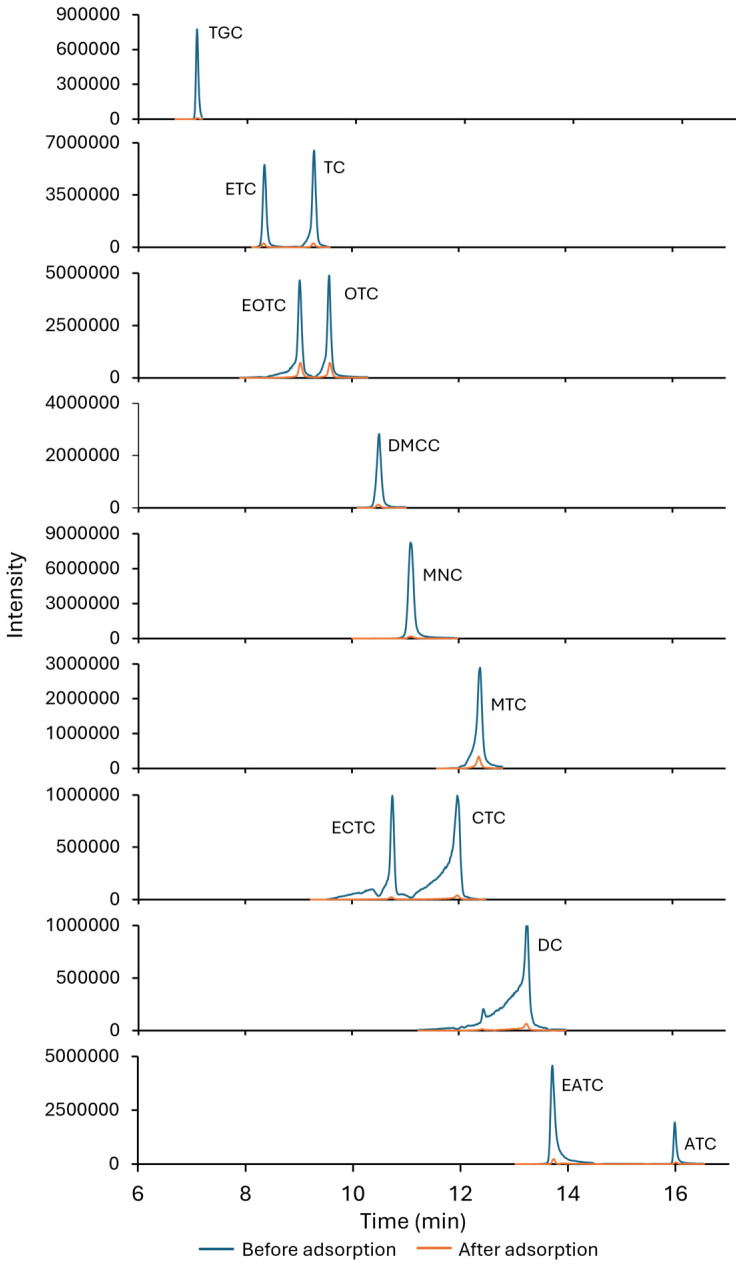
MRM chromatograms of an influent wastewater sample spiked at 2 mg L^−1^ before and after adsorption onto ST.

The proposed adsorbent exhibited an exceptionally high adsorption efficiency (>99%) for all tetracycline antibiotics and their metabolites, even in complex environmental matrices (Figure S10). However, a slight reduction in the adsorption capacity was observed for OTC and MTC (from 99.98% to 99.82 and 99.98% to 99.9%, respectively), with a progressive decline as the matrix complexity increased, following the trend of distilled water > tap water > surface water > effluent wastewater > influent wastewater. Figure 9 presents the MRM chromatogram of an influent wastewater sample, displaying peaks corresponding to the different compounds and their epimers (metabolites), which typically exhibit shorter retention times under the conditions described in Section 3.5. Since epimers share the same transitions, they are shown together in the chromatogram. The figure allows a direct comparison of peak intensities between an influent wastewater sample spiked at 2 mg L^−1^ and the same sample after undergoing the adsorption process with ST. A notable reduction in peak intensities is observed, indicating a substantial decrease in the antibiotic concentration. This confirms that nearly all tested compounds are efficiently adsorbed by the clay in real samples, demonstrating its high removal capacity.

Previous studies have extensively investigated the potential of clay minerals in water treatment, particularly for the removal of heavy metals and organic pollutants. Recently, attention has shifted towards their use for contamination with pharmaceuticals, including tetracyclines. Several studies have evaluated the efficiency of natural and modified clay materials for the adsorption of tetracyclines under different environmental conditions. Table 3 summarizes studies that have investigated the adsorption of these antibiotics onto natural clays, aligning with the present study.

These studies differ in terms of the material dosage used and the concentrations of tetracyclines tested, which could influence the adsorption capacity and the results obtained. Notably, most studies focus on a single antibiotic, TC. For example, Zhao et al. (2012) investigated the adsorption of TC onto montmorillonite and assessed the influence of cations and humic acid on the process. Their findings revealed a high maximum adsorption capacity for TC (250 mg g^−1^) and an equilibrium time of 24 h. Monovalent and divalent cations exhibited distinct effects on the adsorption behaviour [39]. Similarly, Ortiz-Ramos et al. (2022) studied the removal of TC from aqueous solutions through adsorption onto raw Ca-bentonite. The results showed that bentonite achieved a high adsorption capacity (283.5 mg g^−1^) that was comparable to that of carbon-based materials. The high capacity was attributed to cation exchange mechanisms and electrostatic interactions between TC and the bentonite surface [40]. Only Antón-Herrero et al. (2018) studied the adsorption of three tetracyclines (CTC, TC, and OTC) onto ST and biochars, demonstrating a high adsorption capacity on the clay, with a maximum of 140 mg g^−1^ [16]. Compared to these previous studies, the equilibrium time in the present work was significantly shorter, reducing it from several hours to just minutes. Nearly complete equilibrium was reached within only 30 min, with over 99% of adsorption occurring within the first 5 min, indicating a high adsorption capacity.

Other studies have also explored these minerals with certain modifications to enhance the adsorption efficiency. For instance, Wang et al. (2021) investigated the removal of OTC using organo-modified bentonite [41]. The results showed that the introduction of hexadecyltrimethylammonium bromide significantly enhanced the adsorption efficiency, increasing the removal capacity from 75 mg g^−1^ (natural bentonite) to 126 mg g^−1^. The study highlighted the role of hydrophobic interactions and electrostatic attraction in improving adsorption performance. To our knowledge, no data have been reported addressing the adsorption of a broad group of tetracyclines and their metabolites using clay-based materials. A distinctive feature of this study is the comparison of the adsorption efficiencies under real environmental conditions, as most research has focused on batch experiments using distilled water. These results highlight not only the high adsorption efficiency of the clay material used but also a possible synergistic effect among all the compounds, which contributes to maintaining high removal rates even under complex conditions.

**Table 3:** Comparative study of the adsorption of tetracyclines onto natural clays: mineralogical group, adsorption capacity, equilibrium time, and removal efficiency.

Clay Material	Tetracycline(s)	Adsorbent Dose (g L^−1^)	Tetracycline Concentration (mg L^−1^)	q_max_ (mg g^−1^)	% Adsorption	Equilibrium Time	References
Kaolinite group							
Kaolinite	TC	4 1	50	47	90%; ~100%	4 h; 24 h	[42]
Kaolinite	TC	1	0.113 mM	-	90%	24 h	[43]
Illite group							
Illite	TC	10	1000	32	-	8 h	[44]
Vermiculite group							
Vermiculite	OTC	2	160	37	77%	24 h	[45]
Palygorskite group							
palygorskite	TC	0.5	200	99	93%	2 h	[46]
Smectite group							
Montmorillonite	TC	0.2	0.113 mM	-	90%	24 h	[43]
Montmorillonite (SWy-2)	TC	0.5	250	227	70.76%; 78.64%	0.25 h; 6 h	[47]
Montmorillonite	TC	0.2	100	250	~100%	24 h	[39]
Montmorillonite	TC	25	450	287	>85%	24 h	[35]
Ca-bentonite	TC	0.2	250	284	-	7 days	[40]
Bentonite	TC	1.25	60	537	-	8 days	[48]
Bentonite	TC	0.4	200	157	<60%	4.2 h	[31]
Maknessy–Mazzouna	TC	50	1000 µM	368	~100%	24 h	[49]
Stevensite	OTC, TC, and CTC	5	1000	126–140	~100%	24 h	[16]
Stevensite	TC, OTC, CTC, DC, MTC, MNC, DMCC, TGC, ETC, ATC, EATC, EOTC, and ECTC			>86 (for all compounds)	>99%	0.5 h	present study

## 3. Materials and Methods

### 3.1. Chemicals and Reagents

High-purity standards of MTC, DMCC, and ATC were procured from Toronto Research Chemicals (Toronto, ON, Canada). Additional standards, including TGC, EATC, CTC, DC, MNC, TC, and OTC, were purchased from Sigma-Aldrich (Steinheim, Germany). Acros Organics (Geel, Belgium) supplied ETC, ECTC, and EOTC. The purity of most standards exceeded 95%, except for CTC, which had a reported purity of >75%. The Supplementary Materials (Table S3) include detailed information on the molecular structures, CAS numbers, molecular weights, as well as physicochemical properties, such as the pK_a_ and log K_ow_, of the studied compounds.

Other reagents used included humic acid and ammonium formate (≥99.0%) from Sigma-Aldrich (Madrid, Spain). Formic acid (≥99.0%), sodium chloride, hydrochloric acid, and sodium hydroxide were supplied by Panreac (Barcelona, Spain). LC-MS-grade solvents, such as water, methanol, acetone and acetonitrile, were obtained from Merck (Darmstadt, Germany). A stock solution of all tetracycline standards was prepared at a concentration of 1000 mg L^−1^ in methanol. Working solutions containing all the analytes were made by diluting the stock solution with ultrapure deionized water, minimizing the methanol content to reduce interference from organic co-solvents. Stock solutions were stored at −20 °C, while working solutions were kept refrigerated at 4 °C.

The clay utilized in this study was Rhassoul, a Moroccan lava clay found in the Jebel Rhassoul region located within the Atlas Mountains of Morocco. Its composition is predominantly ST, with the following oxide percentages: 69.048% SiO_2_, 18.878% MgO, 4.656% Al_2_O_3_, 0.397% TiO_2_, 1.360% CaO, 1.291% K_2_O, 0.216% Na_2_O, 0.573% P_2_O_5_, 0.014% Cr_2_O_3_, 0.191% SrO, 0.098% SO_3_, 3.251% Fe_2_O_3_, and 0.026% MnO [11].

### 3.2. Purification and Pretreatment of Stevensite (ST)

First, the raw clay was subjected to a washing process using distilled water, followed by drying, milling, and sieving. The clay was then processed following the methodology outlined by Ben Seddik et al. (2019) [17]. A total of 30 g of the washed clay was mixed with 1 L of distilled water and stirred for 30 min. To remove carbonates, drops of 1 M HCl were gradually added until CO_2_ release ceased. The decarbonated material was then treated to obtain the Na^+^ homoionic form by dispersing the powder in a 1 M NaCl solution, stirring for 12 h, and separating the solid by centrifugation.

To isolate fine particles smaller than 2 µm, the resulting Na^+^-ST suspension was transferred to a 2 L test tube, where particle separation was achieved using Stokes’ law. The purified material was then dried at 50 °C for 24 h and subsequently ground into a fine powder using a mortar.

### 3.3. Characterization Analysis

The adsorbent was characterized before and after the adsorption experiments using several analytical techniques: SEM/EDS, XRD, specific surface area analysis, zeta potential measurements, and FTIR. All analyses, except FTIR, were conducted at the CITIUS Laboratories of the University of Seville, Spain.

The SEM/EDS analysis was performed using an FEI-TENEO scanning electron microscope equipped with an energy-dispersive X-ray microanalysis system from FEI Ltd. (Hillsboro, OR, USA). X-ray diffraction was carried out with a Bruker D8 Advance A25 diffractometer (Bruker, Germany) equipped with a CuKα radiation source (40 kV, 30 mA). Diffractograms were recorded across a 2Ɵ range of 1–70° using a step size of 0.03° and a step time of 0.1 s. FTIR spectroscopy was conducted with a Cary 630 FT-IR spectrometer (Agilent Technologies, Santa Clara, CA, USA) over a spectral range of 4000–400 cm^−1^ at a resolution of 4 cm^−1^. The specific surface area and pore volume were determined using N_2_ adsorption isotherms obtained with an ASAP 2420 gas sorption analyser (Micromeritics Instrument Corp., High Wycombe, UK). Measurements were taken at 77 K after the adsorbent was degassed at 373 K for 3 h.

The zeta potential and particle size were measured using a Zetasizer Nano system (Malvern Instruments, Malvern, UK). A suspension of the adsorbent was prepared at a concentration of 0.2 g L^−1^ in water and stirred for 24 h to ensure equilibrium. The pH of the suspension was measured and adjusted as needed using fresh samples, with 0.1 M HCl or NaOH solutions used for pH adjustment. a BASIC 20 pH meter (Crison Instruments, Barcelona, Spain) was used for pH measurements. Zeta potential measurements were performed at 25 °C for both equilibrium and non-equilibrium suspensions.

### 3.4. Batch Adsorption Experiments

Batch adsorption experiments were performed using suspensions of the purified ST in aqueous solutions containing the target tetracycline antibiotics at an initial concentration of 2 mg L^−1^. For each experiment, 10 mL of the solution was transferred into 30 mL glass vials, and 20 mg of adsorbent was added to achieve a suspension concentration of 2 g L^−1^. This amount of ST was selected based on preliminary studies and the existing literature to ensure reliable and reproducible results [11,14]. The vials were stirred at 350 rpm using a multi-stirrer magnetic device (J.P. Selecta S.A., Barcelona, Spain) at room temperature for 24 h to ensure thorough contact between the adsorbent and the antibiotics. The experiments assessed environmental factors, adsorption kinetics, isotherms, adsorbent reusability, and its applicability to real water matrices. All conditions were held constant except for the variable being evaluated in each case.

To investigate environmental effects, the influences of pH (ranging from 2 to 9), salinity (1% to 4%), and dissolved organic matter (0.5–10 mg L^−1^ of humic acid) were examined. pH adjustments were made using 0.1 M HCl or NaOH. Adsorption kinetics were evaluated under optimal pH conditions, with contact times varying from 5 s to 24 h. Isotherm studies were conducted using adsorbate concentrations ranging from 0.25 mg L^−1^ to 100 mg L^−1^. Desorption and reusability tests were performed using 5 mL of a McIlvaine buffer and acetone mixture (7:3, *v*/*v*) at pH 4. After each adsorption cycle, the solid phase was separated by centrifugation at 2260 g for 10 min, treated with 5 mL of desorption solvent, washed with 5 mL of deionized water, and oven-dried at 40 °C. Aliquots of the desorption solvents were filtered and analysed, and the adsorbent was reused for up to five adsorption–desorption cycles with the most effective solvent.

The adsorbent’s performance was further tested in real water samples spiked with 2 mg L^−1^ of tetracycline antibiotics. The water matrices included distilled water, tap water, river surface water, and influent and effluent wastewater. The experimental conditions are summarized in Table S4. To account for potential losses due to degradation, procedural controls without the adsorbent material were included. All experiments were conducted in duplicate. After adsorption, samples were filtered through 0.2-micron filters, diluted with deionized water if necessary, and analysed by liquid chromatography–tandem mass spectrometry (LC-MS/MS).

### 3.5. LC-MS/MS Analysis

The chromatographic analysis was carried out using an Agilent 1290 Infinity II LC system (Agilent Technologies, Santa Clara, CA, USA), which included a vacuum degasser, binary pump, and an automatic injector. Separation was performed on a Zorbax RRHD Eclipse Plus C18 column (150 mm × 3.0 mm i.d., 1.8 μm particle size) (Agilent, USA) maintained at 35 °C. The column was protected by a Zorbax RRHD Eclipse Plus C18 guard column (3.0 mm i.d., 1.8 μm particle size) (Agilent, USA). A 5 µL injection volume was used for all analyses.

The mobile phase consisted of two components: solvent A (10 mM ammonium formate buffer containing 0.1% formic acid and 0.5 mM ammonium fluoride) and solvent B (methanol). The flow rate was set to 0.3 mL min^−1^, and the gradient elution program was as follows: 10% solvent B for the first 5 min, followed by a linear increase in the amount of solvent B to 40% over 1 min (held for 2 min). The amount of solvent B increased to 48% in 1.5 min (held for 3 min) and finally to 100% in 0.5 min (and held for 5 min). Re-equilibration to the initial conditions (10% solvent B) was achieved over 1 min and maintained for 4 min. The total run time was 23 min.

The LC system was coupled to an Agilent 6495 triple quadrupole mass spectrometer (QQQ) equipped with electrospray ionization (ESI) source. The ionization parameters were as follows: capillary voltage, 4000 V; nebulizer pressure, 40 psi; drying gas flow rate, 11 L min^−1^; drying gas temperature, 350 °C; sheath gas flow rate, 12 L min^−1^; sheath gas temperature, 250 °C; and fragmentor voltage, 166 V.

The mass spectrometric analysis was conducted in Dynamic Multiple Reaction Monitoring (dMRM) mode under positive ionization. For each analyte, two transitions were monitored: the most abundant transition (MRM1) was used for quantification, while the secondary transition (MRM2), along with the MRM1/MRM2 ratio, was used for confirmation. The system was operated, and the data were processed using Agilent’s MassHunter Quantitative Analysis 10.1 software (Agilent Technologies, Santa Clara, CA, USA).

Detailed chromatographic conditions, detection parameters, and a detailed list of MS transitions for each compound are provided in the Supplementary Materials (Table S5).

### 3.6. Data Analysis

The removal capacity of tetracycline antibiotics by the purified ST adsorbent was calculated using Equations (1) and (2), respectively:

(1) $$R=\frac{\left(C_{0}-C_{e}\right)}{C_{0}}\times 100$$

(2) $$q_{t}=\frac{\left(C_{0}-C_{e}\right)\;V}{m}$$

In these equations, *R* represents the percentage of tetracyclines removed, while *q_t_* indicates the amount of tetracyclines adsorbed (mg g^−1^). *C*_0_ is the initial adsorbate concentration (mg L^−1^), *C_e_* is the equilibrium concentration of the adsorbate (mg L^−1^), *V* is the solution volume (0.01 L), and *m* is the adsorbent mass (0.02 g).

Adsorption kinetics were evaluated by fitting the experimental data to two kinetic models: PFO and PSO [30,31,32,33]. The adsorption isotherm data were analysed using the Langmuir and Freundlich models. Nonlinear optimization techniques, implemented in Excel Solver, were used to calculate the parameters for each model, as described by Tran et al. (2017) [50]. The goodness of fit for the models was assessed through the normalized root mean square error (NRMSE), chi-square (χ^2^), and the coefficient of determination (R^2^). The equations for the models are provided in Table S2.

## 4. Conclusions

In this work, ST was evaluated as a cost-effective alternative adsorbent for the removal of tetracycline antibiotics and their major metabolites from contaminated water. The findings highlight the potential of this low-cost clay-based material for the efficient decontamination of tetracyclines from aqueous environments. In addition to being an economical and environmentally friendly adsorbent, its use offers significant advantages, particularly its rapid and high adsorption capacity for tetracyclines, which is attributed to its mesoporous structure and surface characteristics.

A comprehensive physicochemical characterization of ST was performed using SEM/EDS, specific surface area analysis, zeta potential, XRD, and FTIR spectroscopy. Adsorption experiments were conducted to investigate the kinetics and isotherms governing tetracycline uptake. The PSO model provided the best fit for the kinetic data, while the Freundlich isotherm exhibited the highest correlation with the experimental results. However, the findings suggest that both Freundlich and Langmuir isotherm models may explain the adsorption process on the ST surface, indicating the potential for monolayer and multilayer adsorption, depending on the prevailing conditions. The maximum monolayer adsorption capacity (q_max_) was notably high, up to 200,000 mg g^−1^ for TGC.

The adsorption efficiency was significantly influenced by pH, with the highest tetracycline adsorption observed in the pH range of 3 to 6, where these compounds predominantly exist in their zwitterionic form (H_2_TC^0^). Other environmental factors, such as dissolved DOM, had minimal impacts, preserving the strong adsorption performance. However, increased salinity reduced the adsorption efficiency, with up to a 50% decrease observed for OTC. This effect can be attributed to competitive adsorption at active sites and the complexation between sodium ions and tetracycline molecules. The reusability of ST was assessed through five consecutive adsorption–desorption cycles, demonstrating a sustained high sorption capacity, with the retention exceeding 90% after the last cycle in all cases. The characterization of ST before and after batch adsorption experiments confirmed pore filling as a contributing mechanism. The primary adsorption mechanism involves electrostatic interactions, although hydrogen bonding and π-π interactions may also play dominant roles.

The remarkable adsorption capacity of ST for tetracyclines, owing to its physicochemical properties, establishes it as a promising alternative for the removal of these antibiotics from contaminated water and wastewater sources. This approach represents a sustainable and environmentally friendly strategy for water decontamination. Nevertheless, further research is necessary to scale up the process to a pilot plant level and to conduct dynamic adsorption studies using column experiments. Additionally, more in-depth investigations into the potential interactions between the material and complex environmental matrices should be considered.

## Figures and Tables

**Table 1:** Kinetic parameters for the adsorption of tetracyclines onto ST.

Kinetic Parameters	TGC	ETC	EOTC	TC	OTC	DMCC	ECTC	MNC	CTC	MTC	DC	EATC	ATC
Pseudo-first order:													
*k*_1_ (min^−1^)	0.026	0.380	0.463	0.532	0.423	0.274	0.467	0.321	0.452	0.390	0.492	0.165	3.946
	±0.015	±0.002	±0.003	±0.005	±0.008	±0	±0.002	±0	±0.019	±0.006	±0.007	±0.002	±0.004
q^e^_cal_ (mg·g^−1^)	0.063	0.046	0.140	0.221	0.290	0.056	0.26	0.013	0.285	0.155	0.229	0.012	0.048
	±0.004	±0.002	±0.007	±0.004	±0.04	±0	±0.18	±0	±0.06	±0.011	±0.008	±0.002	±0.003
q^e^_exp_ (mg·g^−1^)	1.03	1.05	1.05	1.07	1.29	1.28	0.983	1.14	1.32	1.58	1.17	1.09	0.991
R^2^	0.259	0.738	0.871	0.868	0.879	0.600	0.986	0.717	0.493	0.774	0.962	0.436	0.596
Pseudo-second order:													
*k*_2_ (g·mg^−1^·min^−1^)	19.9	21.4	11.1	56.0	6.2	25.8	11.5	3.84	13.0	6.88	8.65	9.76	2.11
	±0.13	±0.63	±1.8	±12	±1.9	±0	±2	±0.02	±2	±1.2	±1.3	±1.0	±1.1
q^e^_cal_ (mg·g^−1^)	1.034	1.0500	1.051	1.066	1.280	1.280	0.983	1.138	1.317	1.5750	1.167	1.085	0.990
	±0	±0.0002	±0	±0	±0.0013	±0	±0	±0	±0	±0.001	±0.001	±0.0004	±0.005
q^e^_exp_ (mg·g^−1^)	1.034	1.051	1.052	1.067	1.288	1.280	0.983	1.138	1.317	1.575	1.168	1.086	0.991
R^2^	1.0000	1.0000	1.0000	1.0000	1.0000	1.0000	1.0000	1.0000	1.0000	1.0000	1.0000	1.0000	1.0000

**Table 2:** Adsorption isotherm parameters for the adsorption of tetracyclines onto ST.

Model Parameters	TGC	ETC	EOTC	TC	OTC	DMCC	ECTC	MNC	CTC	MTC	DC	EATC	ATC
Langmuir:													
q_max_ (mg·g^−1^)	86.1	856.3	1000	1000	1000	1000	221.6	262.7	625.6	609.2	777.2	851.1	883.6
*K*_L_ (mg·g^−1^)	0.029	0.002	0.001	0.001	0.001	0.001	0.007	0.007	0.003	0.002	0.002	0.002	0.002
R^2^	0.863	0.829	0.813	0.807	0.755	0.817	0.842	0.881	0.893	0.817	0.814	0.908	0.905
Freundlich:													
*K*_F_ (mg^1−^^*n*^·L*^n^*·g^−1^)	12.0	11.0	10.6	10.4	9.8	10.9	11.3	12.0	11.8	10.8	10.7	11.8	11.7
*n*	0.325	0.320	0.315	0.323	0.288	0.313	0.321	0.349	0.369	0.311	0.314	0.389	0.385
R^2^	0.968	0.893	0.857	0.817	0.864	0.883	0.926	0.940	0.911	0.891	0.864	0.931	0.928

## Data Availability

All data generated for this study are contained within the article and Supplementary Materials.

## Supplementary Materials

The following supporting information can be downloaded at https://www.mdpi.com/article/10.3390/antibiotics14040395/s1, Table S1. Characterization of ST before and after adsorption; Table S2. Kinetic models and adsorption isotherms studied; Table S3. Physical–chemical properties of the tetracyclines and their metabolites; Table S4. Conditions applied in the batch experiments; Table S5. LC-MS/MS parameters employed in the determination of tetracyclines and their metabolites; Figure S1. N_2_ adsorption/desorption isotherms before (top panel) and after (bottom panel) ST adsorption; Figure S2. BET surface area of ST before and after tetracycline adsorption; Figure S3. SEM images of ST before adsorption (left panel, 3000× and 6000×) and after tetracycline adsorption (right panel, 700× and 3000×) showing an entire particle and the surface of the material; Figure S4. Kinetics of tetracycline adsorption onto ST; Figure S5. Pseudo-first-order kinetic model for tetracycline adsorption onto ST: graphical determination of rate constants (ln(qe-qt) vs. time); Figure S6. Pseudo-second-order kinetic model for tetracycline adsorption onto ST: graphical determination of rate constants (t/qt vs. time); Figure S7. Effect of dissolved organic matter on the adsorption (%) of tetracyclines on ST; Figure S8. Effect of the salt content (NaCl, % *w*/*v*) on the adsorption (%) of tetracyclines on ST; Figure S9. Percentage (%) of tetracyclines adsorption on ST in the evaluation of reuse after five consecutive cycles; Figure S10. Influence of real environmental matrices on the adsorption of tetracyclines onto ST. References [51,52,53] are cited in the Supplementary Materials.

## Abbreviations

ATC	anhydrotetracycline
CTC	chlortetracycline
C_e_	equilibrium concentration
DC	doxycycline
DMCC	demeclocycline
dMRM	dynamic multiple reaction monitoring
DOM	dissolved organic matter
EDS	energy-dispersive X-ray spectroscopy
EATC	4-epianhydrotetracycline
ECTC	4-epichlortetracycline
EOTC	4-epioxytetracycline
ESI	electrospray ionization
ETC	4-epitetracycline
FTIR	Fourier transform infrared spectroscopy
*k*_1_	PFO kinetic constants
*k*_2_	PSO kinetic constants
*K*_F_	Freundlich constant (L mg^−1^)
*K*_L_	Langmuir dissociation constant (L mg^−1^)
LC-MS/MS	liquid chromatography coupled to tandem mass spectrometry
MNC	minocycline
MTC	metacycline
*n*	Freundlich constant related to the adsorption strength of the adsorbent
NRMSE	normalized root mean square error
OTC	oxytetracycline
PFO	pseudo-first order
PSO	pseudo-second order
q	equilibrium adsorption capacity (mg g^−1^)
q_e_	amount of tetracyclines adsorbed at equilibrium (mg g^−1^)
q_max_	maximum amount adsorbed within a monolayer (mg g^−1^)
q_t_	amount of tetracyclines adsorbed at determined time (mg g^−1^)
QQQ	triple quadrupole mass spectrometer
SEM	scanning electron microscopy
ST	stevensite
TC	tetracycline
TGC	tigecycline
XRD	X-ray diffraction
