# Supplementary material for: Efficient Removal of Tetracyclines and Their Metabolites from Wastewater Using Purified Stevensite: Adsorption Capacity, Reusability, and Antibiotic Decontamination
Source: Antibiotics (Basel). 2025 Apr 10;14(4):395. doi: 10.3390/antibiotics14040395 (PMC12024152; doi:10.3390/antibiotics14040395)
Supplement: Supplementary file 1 [file antibiotics-14-00395-s001.zip › antibiotics-3551608-supplementary.pdf]

Supplementary material

Efficient Removal of Tetracyclines and Their Metabolites from Wastewater Using Purified Stevensite: Adsorption Capacity, Reusability, and Antibiotic Decontamination

Noelia García-Criado, Laura Martín-Pozo, Julia Martín*, Juan Luis Santos, Irene Aparicio, Esteban Alonso

Departamento de Química Analítica, Escuela Politécnica Superior, Universidad de Sevilla. E-41011 Seville, Spain; ngarcia5@us.es (N.G.-C); lpozo@us.es (L.M.-P.); jbueno@us.es (J.M.); jlsantos@us.es (J.L.S.); iaparicio@us.es (I.A.); ealonso@us.es (E.A.).

Corresponding author: Julia Martín Bueno

Email: jbueno@us.es

Table S1. Characterization of ST before and after adsorption.

	Z potential	Surface area ($m^2\ g^{-1}$)	Pore volume ($cm^3\ g^{-1}$)	Pore size (nm)
ST before adsorption	-8.65	146.89	0.1218	3.3156
ST after adsorption	-8.14	110.98	0.0914	3.2955

Table S2. Kinetic models and adsorption isotherms studied.

Kinetic	Model equation
Pseudo-first order (PFO)	$\ln (q_e-q_t) = \ln q_e-k_1 \times t$<br>$q_e$ and q_t: amounts of compounds (mg/g) adsorbed at equilibrium and at a t time;<br>t: time (min);<br>k_1: PFO kinetic constants.
Pseudo-second order (PSO)	$t/q_t = 1/(k_2 \times q_e^2) + t/q_e$<br>$q_e$ and q_t: amounts of compounds (mg/g) adsorbed at equilibrium and at a t time;<br>t: time (min);<br>k_2: PSO kinetic constants.

Isotherm	Model Equation
Langmuir	$q = \frac{q_{max}K_L C_e}{1+(C_e K_L)}$<br>$C_e$: Equilibrium concentration of pollutants (mg/L);<br>q: Equilibrium adsorption capacity (mg/g);<br>q_{max}: maximum amount adsorbed within a monolayer (mg/g);<br>K_L: Langmuir dissociation constant (L/mg), which is related to the adsorption energy.
Freundlich	$q = K_F C_e{}^{1/n}$<br>C_e: Equilibrium concentration of pollutants (mg/L);

	q: Equilibrium adsorption capacity (mg/g);<br>K_F: Freundlich constant (L/mg), which is related to the affinity of the adsorbent to the adsorbate;<br>$1/n$: dimensionless parameter, which indicates how adsorption varies as a function of the concentration.

Table S3. Physical-chemical properties of the tetracyclines and their metabolites.

Tetracyclines	Acronym	MW	pKa	Log K_{OW}	Molecular formula
Tetracycline	TC	444.44	3.30[a]/7.68[b]/ 9.69[b]	-1,30[a]	$C_{22}H_{24}N_2O8$
4-Epitetracycline	ETC	444.15	4.8, 8.0[c]	-1.33[b]	$C_{22}H_{24}N_2O8$
Anhydrotetracycline	ATC	426.14			$C_{22}H_{22}N_2O_7$
4-Epianhydrotetracycline	EATC	426.14			$C_{22}H_{22}N_2O_7$
Chlortetracycline	CTC	478.88	2,99[a]/7.4[b]/ 9.3[b]	-0.62[b], -0.36[b], -2,9[a]	$C_{22}H_{23}ClN_2O_8$
4-Epichlortetracycline	ECTC	478.11			$C_{22}H_{23}ClN_2O_8$
Oxytetracycline	OTC	460.43	3.27[a]/7.3[b]/ 9.1[b]	-0.90[a], -1.22[b]	$C_{22}H_{24}N_2O_9$
4-epioxytetracycline	EOTC	460.15			$C_{22}H_{24}N_2O_9$
Metacycline	MTC	478.11	3.5/7.6/9.2[c]	-0,3[a]	$C_{22}H_{23}ClN_2O_8$
Doxycycline	DC	444.15	3.09[a]/7.7[b]/ 9.5[b]	-0.54, 0.69[a]	$C_{22}H_{24}N_2O_8$
Minocycline	MNC	457.18	2.8/5.0/7.8/9.5[d]	0.05[a]	$C_{23}H_{27}N_3O_7$
Tigecycline	TGC	585.28	4.5[a]		$C_{29}H_{39}N_5O_8$
Demeclocycline	DMCC	464.1	3.3/7.2/9.2[b]	-1.14[b]	$C_{21}H_{21}ClN_2O_8$

Spike levels: Metabolites are indicated in italics; abbreviations are written in brackets. MW: molecular weight (g mol^{-1}), pK_a: acid dissociation constant, K_{ow}: octanol-water partition coefficient. References: [51]; [b][52]https://www.chemicalbook.com/; [c][53].

Table S4. Conditions applied in the batch experiments.

Equilibrium time optimisation	
Agitation time (min)	0.083, 0.5, 0.75, 1, 5, 10, 30, 60, 180, 600, 1440
Concentration (mg/L)	2
pH	Not controlled
Salinity (%)	0
Organic matter (mg/L)	0
Aquatic environments	Distilled water
Temperature (°C)	25

Adsorption isotherms	
Agitation time (min)	60
Concentration (mg/L)	1, 2, 5, 10, 15, 20, 50, 100
pH	Not controlled
Salinity (%)	0
Organic matter (mg/L)	0
Aquatic environments	Distilled water
Temperature (°C)	25

Influence of pH	
Agitation time (min)	60
Concentration (mg/L)	15
pH	2, 4, 7, 9
Salinity (%)	0
Organic matter (mg/L)	0
Aquatic environments	Distilled water
Temperature (°C)	25

Influence of salinity percentage	
Agitation time (min)	60
Concentration (mg/L)	15
pH	Not controlled
Salinity (%)	0, 1, 2, 3, 4
Organic matter (mg/L)	0
Aquatic environments	Distilled water
Temperature (°C)	25

Influence of organic matter percentage	
Agitation time (min)	60
Concentration (mg/L)	15
pH	Not controlled
Salinity (%)	0
Organic matter (mg/L)	0, 5, 10, 20, 25
Aquatic environments	Distilled water
Temperature (°C)	25

Influence of different aquatic environments	
Agitation time (min)	60
Concentration (mg/L)	2
pH	Not controlled

Salinity (%)	0
Organic matter (mg/L)	0
Water source	Influent wastewater, effluent wastewater, surface water and tap water
Temperature (°C)	25

Table S5. LC-MS/MS parameters employed in the determination of tetracyclines and their metabolites.

**Chromatographic conditions**	
**Column**	ZORBAX Eclipse Plus C_{18} (3 mm x 150 mm d.i., 1,8 µm)
**Precolumn**	guard column ZORBAX Eclipse plus C_{18} (2.1 mm × 5 mm, 1.8 µm)
**Flow**	0.3 mL·min^{-1}
**Column temperature**	35 ºC
**Analysis time**	21 min
**Mobile phase**	A: Ammonium formate solution 10 mM and ammonium fluoride 0.05 mM (0.1% formic acid)<br>B: Methanol

Elution gradient

Time (min)	0	5	6	8	9.5	12.5	13	18	19	23
Solvent A (% v/v)	90	90	60	60	52	52	0	0	90	90
Solvent B (% v/v)	10	10	40	40	48	48	100	100	10	10

Mass spectrometer conditions

Tetracycline/*metabolite*	Precursor ion *(m/z)*	Product ions (quantifier/ qualifier) (m/z)	Collision Energy (eV)	Ion ratio	Retention time (min)	Ion Polarity
TC	445.2	410.2/154.1	20.0/30.0	71.3	11.44	Positive
ETC	445.1	410.2/98.1	20.0/44.0	34.8	10.63	Positive
ATC	427.2	153.9/410.1	20.0/28.0	49.0	16.83	Positive
EATC	427.2	410.1/321.1	20.0/32.0	10.9	16.15	Positive
OTC	461.2	426.1/443.0	20.0/12.0	38.2	11.73	Positive
EOTC	461.2	426.2/444.0	20.0/16.0	71.3	11.14	Positive
CTC	479.1	444.0/462.0	20.0/20.0	64.0	14.18	Positive
ECTC	479.0	444.0/98.0	20.0/42.0	82.2	12.92	Positive
MTC	443.0	426.0/381.0	25.0/25.0	31.6	14.99	Positive
DC	445.2	428.0/154.0	30.0/30.0	41.8	16.04	Positive
MNC	458.2	441.1/283.1	20.0/48.0	22.4	13.20	Positive
DMCC	465.1	448.1/430.1	16.0/24.0	61.6	12.73	Positive
TGC	293.65	513.3/257	43/25	1.0	6.95	Positive

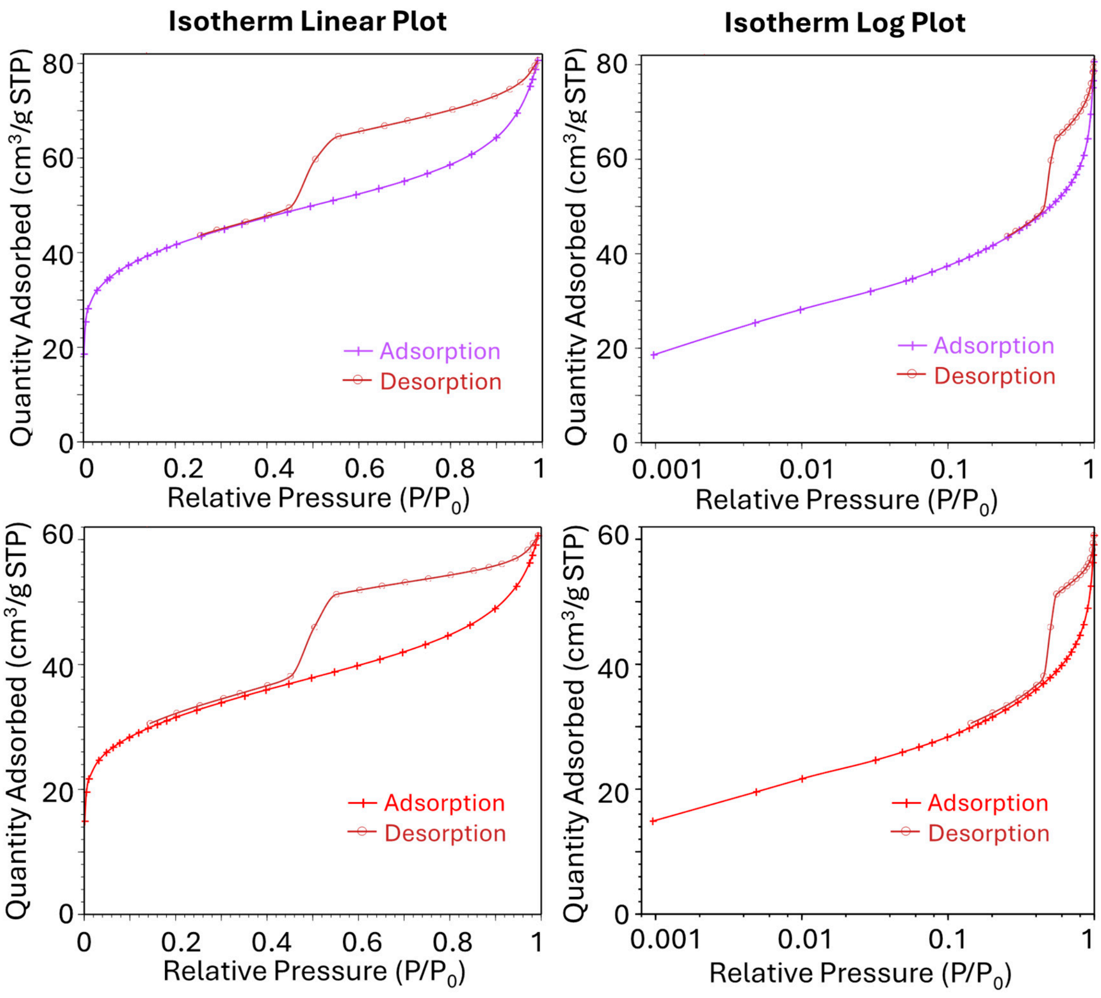

Figure S1. N_2 adsorption/desorption isotherms before (top) and after (bottom) ST adsorption.

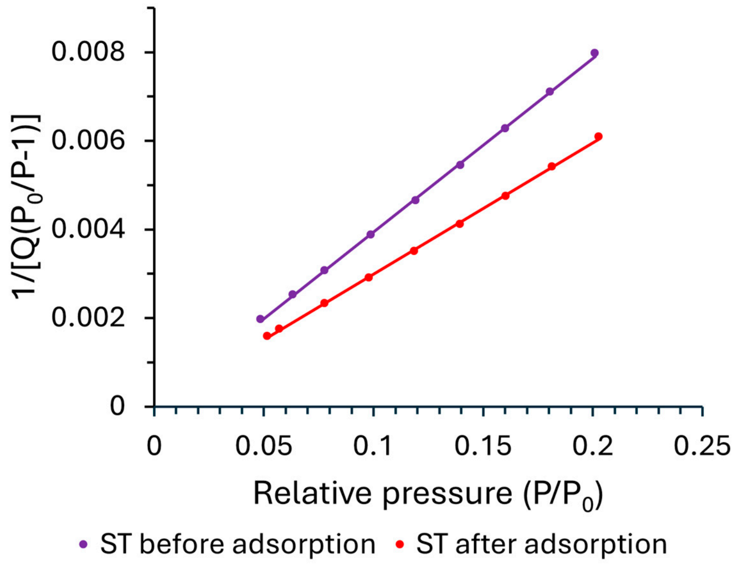

Figure S2. BET Surface area of ST before and after tetracycline adsorption.

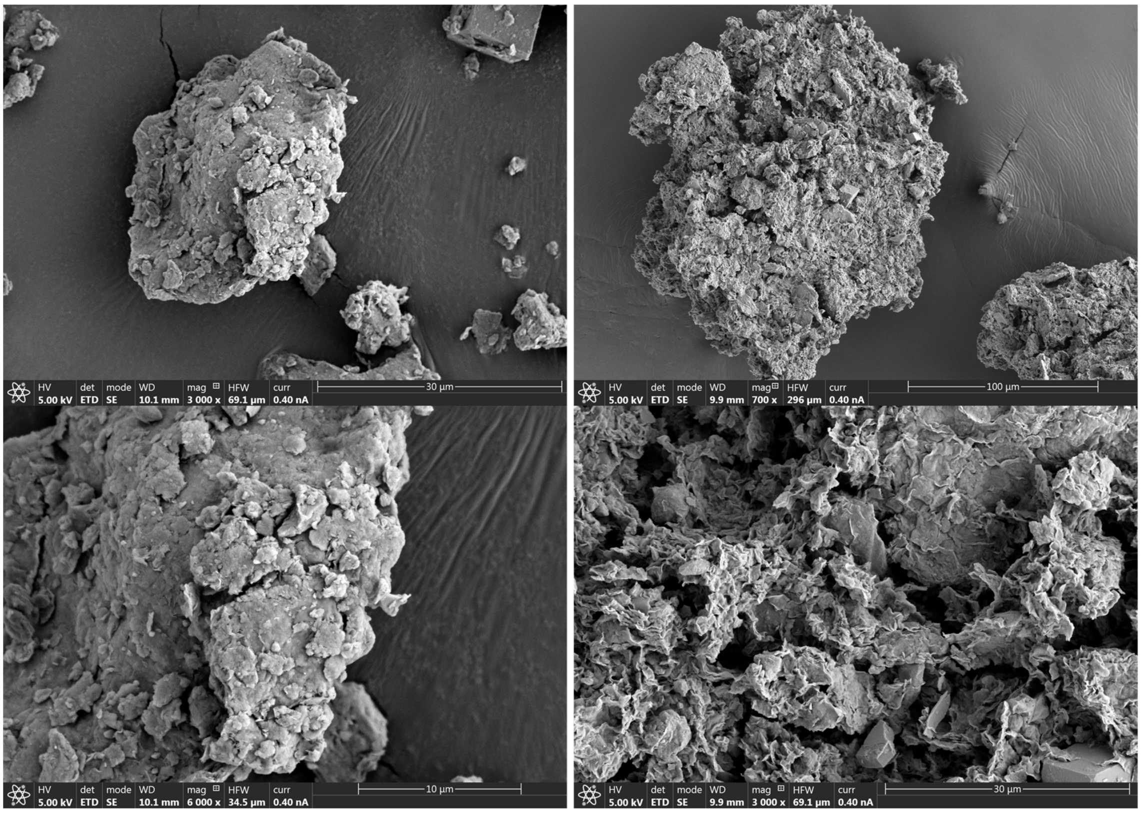

Figure S3. SEM images of ST before adsorption (left, 3000× and 6000×) and after tetracyclines adsorption (right, 700× and 3000×) showing an entire particle and the surface of the material.

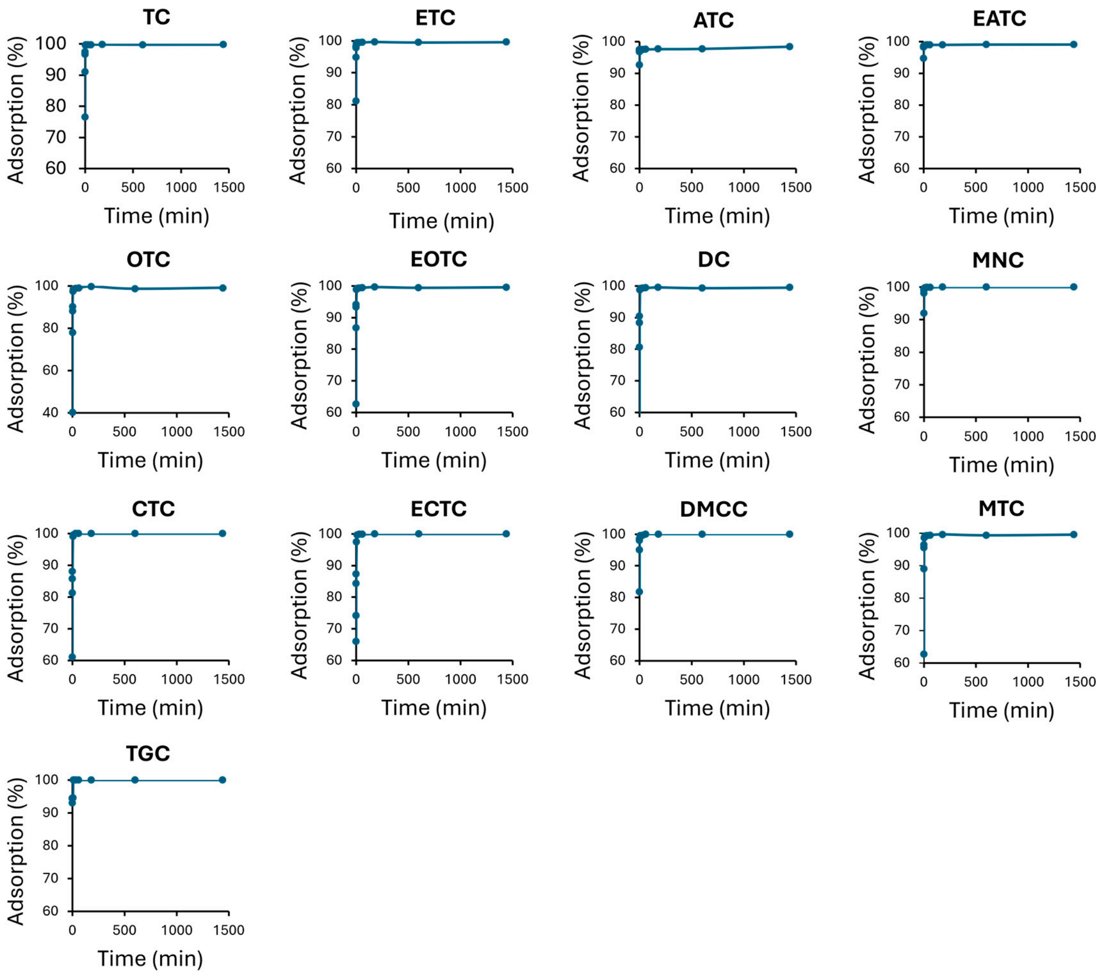

Figure S4. Adsorption kinetic of tetracyclines onto ST.

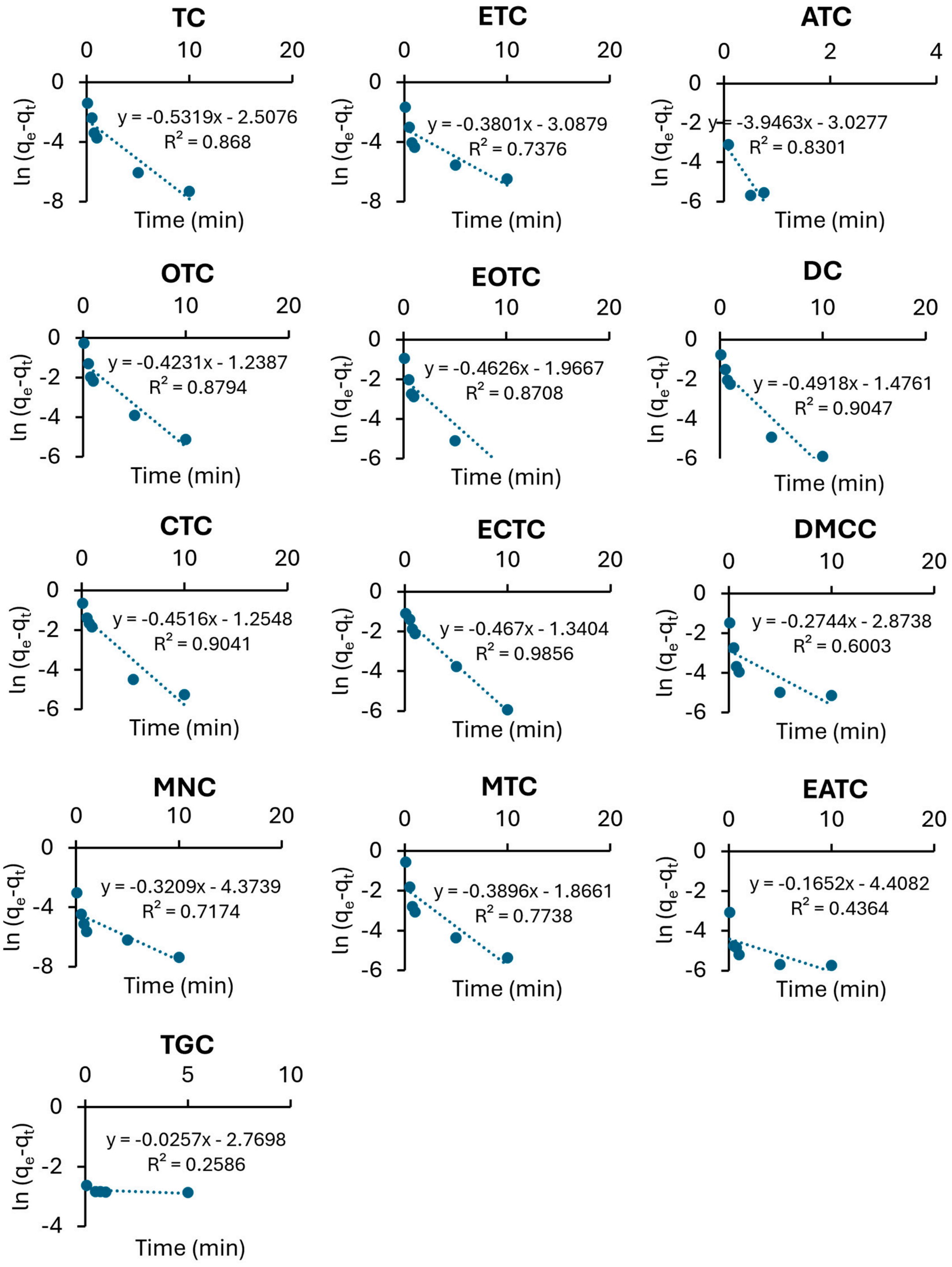

Figure S5. Pseudo-first-order kinetic model for tetracycline adsorption onto ST: graphical determination of rate constants (ln(q_e-q_t) vs. time).

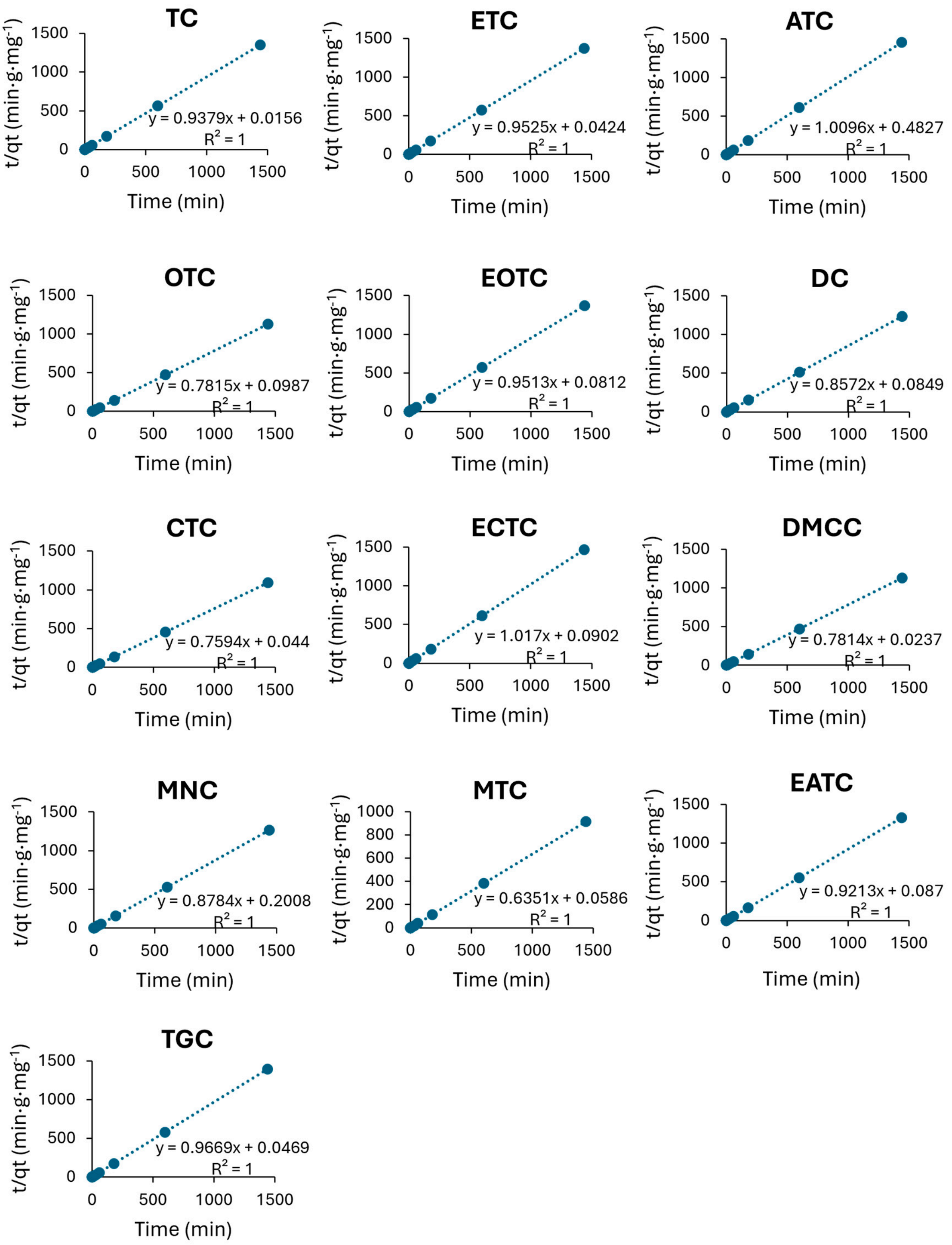

Figure S6. Pseudo-second-order kinetic model for tetracycline adsorption onto ST: graphical determination of rate constants (t/qt vs. time).

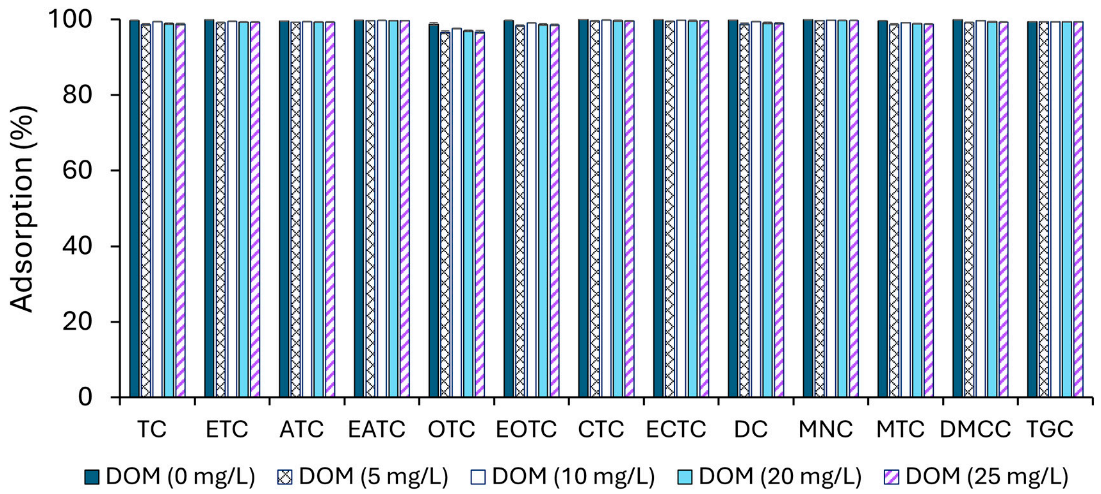

Figure S7. Effect of dissolved organic matter on the adsorption (%) of tetracyclines on ST.

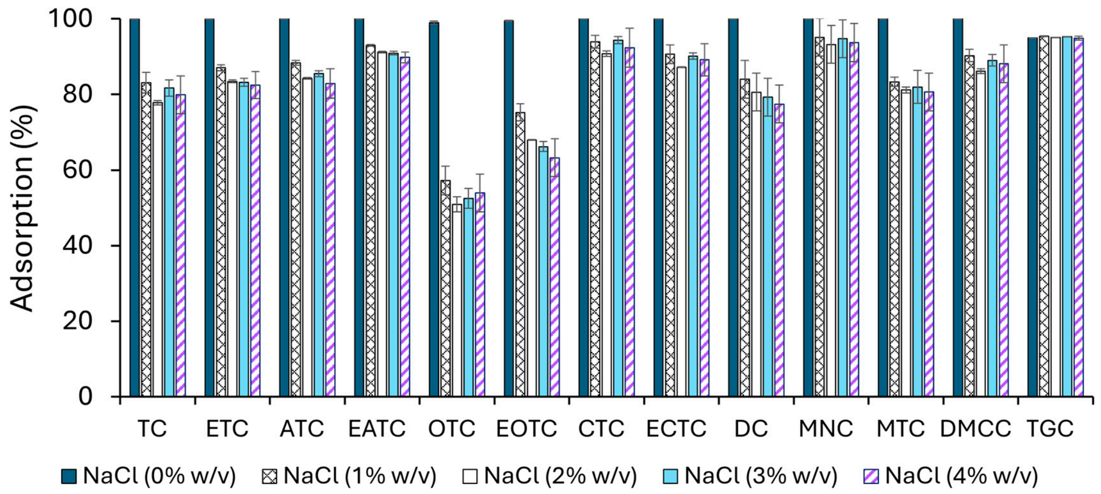

Figure S8. Effect of salinity content (NaCl, % w/v) on the adsorption (%) of tetracyclines on ST.

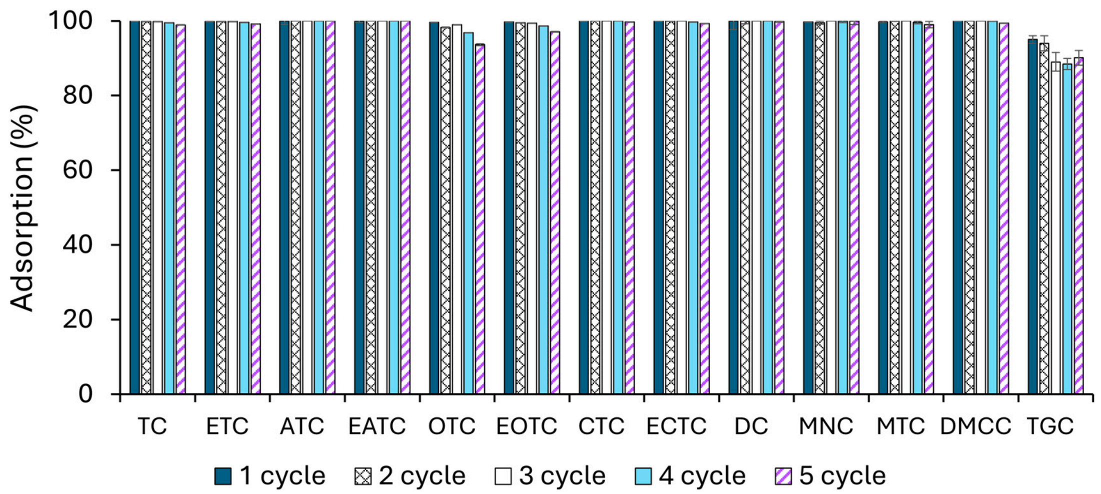

Figure S9. Adsorption percentage (%) of tetracyclines on ST in the evaluation of reuse after five consecutive cycles.

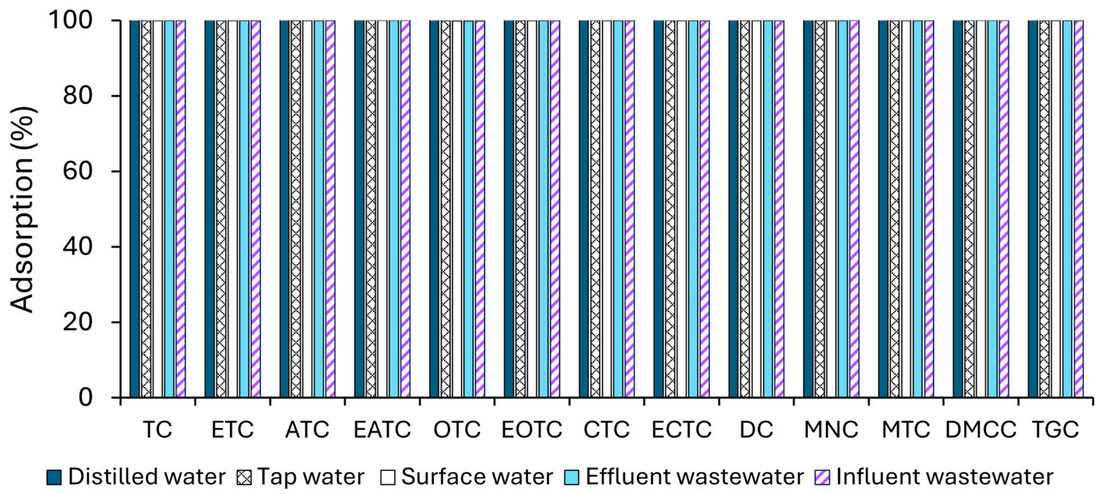

Figure S10. Influence of environmental real matrices on the adsorption of tetracyclines onto ST.
